# Questioning the validity of food addiction: a critical review

**DOI:** 10.3389/fnbeh.2025.1562185

**Published:** 2025-07-30

**Authors:** Jônatas Oliveira, Giulia Cazetta Bestetti, Isis De Carvalho Stelmo, Larissa Antunes, Priscylla Rodrigues Vilella

**Affiliations:** ^1^School of Medicine, University of São Paulo, São Paulo, Brazil; ^2^Institute for Research on Behavior and Food, São Paulo, Brazil; ^3^School of Nutrition, Federal University of Goiás, Goiás, Brazil

**Keywords:** eating disorders, eating behavior, food addiction, food craving, disordered eating

## Abstract

Food cravings (FC) are closely associated with behaviors such as loss of control, binge eating, and emotional eating. Although FC is among the symptoms proposed for food addiction (FA), we argue that the distress associated with eating, managing cravings, and experiencing loss of control may not, in itself, constitute a framework consistent with addiction or addiction-like eating. Grouping these concepts under the FA label may contribute to conceptual confusion, potentially leading to diagnostic inaccuracies. This integrative review aimed to explore the concepts of FA and FC, as well as their interrelations, through methodologies such as self-report questionnaires and visual analog scales. A systematic search without time restrictions yielded 37 original studies for qualitative analysis. The selected investigations examined FA and FC as primary outcomes and were categorized into five thematic sections: (1) genetic factors, (2) neurobiology, (3) behavioral factors, (4) emotional factors, and (5) food cues. The findings indicate that cravings play a mediating role in disordered eating patterns and are associated with excessive consumption or impaired control in individuals exhibiting symptoms attributed to FA. This review addresses three key issues: (i) theoretical and psychometric challenges in the conceptualization of FA, (ii) redundancies among FC, eating-related distress, and self-reported loss of control, and (iii) whether FA is primarily a matter of semantics. Phrases related to constructs of substance use disorders correlate with constructs that are neuropsychopharmacological influenced, and they impose new constructs upon previously established eating behavior patterns, along with their already known neural and neuropsychological correlates (such as emotional eating, dietary restraint, binge eating, and craving). The concept of FA, along with its scale and the new diagnostic questionnaire, also integrates cultural perceptions of food with established psychological constructs, drawing on previously recognized phenomena. Investigating the continuum encompassing (i) cravings, (ii) disordered eating attitudes, and (iii) body image–related distress presents a significant challenge, particularly when researchers overlook the underlying human narratives that define this multifaceted phenomenon. Without a clear theoretical and epistemological framework, the boundaries of FA risk becoming overly broad, diminishing its utility as a diagnostic tool or basis for interventions. The challenges in establishing a consistent and precise definition underscore the need for further research to ensure the concept represents a distinct and scientifically valid phenomenon rather than a generalized reflection of eating-related constructs.

## 1 Introduction

The interplay between food-related behaviors and psychological factors has become a central focus in recent years, particularly with the emergence of the concept of food addiction (FA). FA describes eating behaviors that challenge clinicians and arise during the treatment of various clinical conditions, such as diminished control over consumption, strong cravings, continued use despite negative consequences, and repeated unsuccessful attempts to reduce intake (Hebebrand and Gearhardt, 2021; Schulte and Gearhardt, 2018). Weight and body mass index (BMI) challenge the concept of FA, as it was initially strongly associated with obesity or regarded as a manifestation of binge eating disorder (BED) (Oliveira et al., 2021). Previous studies have already weakened this association (Albayrak et al., 2012). In a nationally representative study, categorization by weight class (underweight, normal weight, overweight, and obesity) was found to be significantly associated with symptom count scores on the Modified Yale Food Addiction Scale 2.0 (mYFAS 2.0). A quadratic relationship was observed, indicating that individuals classified as underweight reported significantly more mYFAS 2.0 symptoms than those in all other weight categories (Schulte and Gearhardt, 2018).

Patients who underwent a preoperative psychological evaluation for bariatric surgery and met the criteria for FA exhibited notably higher levels of binge eating, emotional eating, and lower self-efficacy, highlighting its relevance in identifying psychological comorbidities (Koball et al., 2021). When individuals were interviewed on this topic, they often described a set of recurring experiences and perceptions related to eating behaviors. These include: (1) Reward-driven eating (i.e., eating for psychological rather than physiological reasons), (2) A functional or psychological preoccupation with food, (3) A perceived lack of self-control around food, (4) Frequent cravings, (5) Increased weight or an unhealthy diet, and (6) A problem with a specific type of food (Ruddock et al., 2015). Despite this, all these components are already recognized and treated. What is new is the FA label? Bringing these issues together into a new diagnosis or clinical condition has sparked efforts and debates over the past few years.

An intense desire to consume specific foods, or food cravings (FC), is one of the symptoms included in the diagnostic criteria for FA. Ludwig and Wikler (1974) proposed that “craving functions as a method of protection against distress, alerting the individual to a potential source of relief.” This phenomenon represents a multifaceted experience arising from the intricate connection between emotional, psychological, and biological factors, extending far beyond simple physiological or behavioral responses (Oliveira and Cordás, 2020). They refer to specific motivations that manifest as thoughts and vivid sensations, often accompanied by mental imagery that drives the pursuit and consumption of food for relief and/or pleasure (Kavanagh et al., 2005; May et al., 2015). Emotional states trigger opposing reactions to maintain emotional balance, and stress pathways contribute to these cycles, with internal and external stressors reinforcing maladaptive eating patterns (Parylak et al., 2011). Emotional dysregulation, influenced by cognitive processes like avoidance and fear of weight gain, further complicates craving dynamics (Blackburn et al., 2012). FC and FA have been studied in terms of their predictive value, revealing distinct characteristics when considering positive aspects related to pleasure and consumption (i.e., positive reinforcement) rather than the negative consequences of eating (i.e., loss of control). Meule and Kübler (2012) demonstrated that positive reinforcement associated with FC negatively predicted FA symptoms, whereas other subscales involving distress, guilt, and loss of control showed positive associations with FA. Additionally, FC can be understood through a range of theoretical models (Skinner and Aubin, 2010), which could resemble traditional addiction frameworks, drawing some parallels between disordered eating behaviors, eating disorders (EDs), and substance use disorders (SUD). The definitions of FA and FC are confounded, and lately some propose that the presence of FC, it would indicate one is addicted to food (Malika et al., 2015).

Negative experiences with food consumption are present across different ED diagnosis, as well as those who experience disordered eating. Individuals with Anorexia nervosa (AN) may perceive any food intake as excessive and experience lower levels of FC (Moreno et al., 2009), while those with BED often report a profound sense of loss of control during eating episodes (Adler et al., 2022). This confusion is further compounded by weight stigma narratives, which imply that individuals who do not engage in weight control behaviors must inherently struggle with control, meaning they lack control (Oliveira, 2024). Such assumptions are both false and unscientific. The issue of control is relevant to both AN and BED, as individuals with AN also frequently describe themselves as being “addicted to food” (Song et al., 2024). Patients with AN present a distinctive aspect in the elaboration process of intrusive thoughts during craving experiences. These thoughts are influenced by cognitions and fear of becoming overweight, which induce avoidance and evasion of the appetizing target (Blackburn et al., 2012). Consequently, despite having cravings, they do not always manifest themselves in food intake. These patients typically exhibit a positive result for FA regardless of the presence of binge eating (Granero et al., 2018). In both cases, the subjective and objective experiences of eating can be deeply distressing. Behaviors related to FC reactivity may amplify feelings of impaired control and intensify the sensation that larger quantities are required to achieve satisfaction or conclude a binge episode (Meule and Kübler, 2012; Meule et al., 2014b).

Measures to assess cravings are widely used in research and clinical settings, with advancements aimed at improving reliability and consistency. Meule (2018) identified a score of 50 in a FC questionnaire as an effective threshold for distinguishing FA (Meule, 2018; Haghighinejad et al., 2021). These findings suggest that subjective experiences of loss of control, cognitive associations with food, and the occurrence of FC are key components in understanding FA. If cravings emerge as a response to dietary restrictions and self-imposed limitations, could they partially explain the sensation of loss of control? Similarly, if binge eating episodes exacerbate dysphoria, might these emotional components be mistakenly conflated with addiction? These dynamics highlight the complexity of FA as both a scientific construct and a public health concern. While the addiction framework offers valuable insights into EDs and disordered eating behaviors, it also raises critical questions about what is being measured and how these constructs are understood across different sociocultural contexts. Addressing these questions through further research is essential to refine both the theoretical foundation and the clinical application of FA.

### 1.1 What is food addiction?

FA refers to a condition in which individuals would exhibit behaviors and altered neural patterns similar to those associated with substance use disorders, but they would be triggered by specific types of food, often those high in sugar, fat, and/or salt. According to the APA Dictionary of Psychology (n.d.):

*“An eating disturbance characterized by a preoccupation with one’s body image and weight, obsessive thoughts about food, the use of food as a source of pleasure, and compulsive eating. In addition, the individual may experience symptoms of withdrawal during attempts to reduce food intake or abstain from particular types of food.”*

The concept today is grounded in neurobiological frameworks, emphasizing the role of dopaminergic reward pathways and other systems in compulsive eating behaviors (Teegala et al., 2020; Meule, 2019). For an historic overview we recommend to see the chapter by Meule (2019). Unlike physiological hunger, FA involves an uncontrollable urge to consume specific foods despite adverse consequences. One of the earliest definitions can be found in a document produced by Clouston (1890):

*“It is a fact that some foods are more stimulating to the brain cortex than others, e.g., strong beef-tea than milk, flesh than bread. [.] If from childhood upwards the possessor of such a brain has depended on stimulating diet and drink for its restoration when exhausted, there is an intense and irresistible craving set up for such food and drink stimulants whenever there is fatigue. Such a brain has developed an affinity for them, and for such alone. Milk and farinaceous diet often become repugnant, and when taken do not satisfy the brain craving. Its owner becomes physiologically a flesh-eater and an alcohol-drinker.” (p. 207)*

Subsequently, Wulff (1932) introduced another perspective:

*“I have used the term “eating addiction” above without justifying why I deem it important to call it an “addiction” and not, for example, a compulsion. I believe that the nature of this compulsive eating can be best characterized by the term addiction.” (p. 299)*

In 1956, Theron Randolph described FA as a specific adaptation to regularly consumed foods, producing addiction-like symptoms in highly sensitive individuals:

*“A specific adaptation to one or more regularly consumed foods which, in a highly sensitive person, produces a pattern of symptoms similar to other addictive processes.”*

In 1985, Wurtman, (1985) proposed that carbohydrate consumption acts as a coping mechanism for negative emotions, linking tryptophan intake to serotonin synthesis and suggesting a form of self-medication. This notion remains culturally prevalent today, often expressed when people say they “need chocolate for serotonin.” Animal studies provided the foundation for trials involving palatable foods such as cookies, chocolates, and sweetened water. Hernandez and Hoebel (1988) demonstrated that sucrose solutions stimulate dopamine release in the nucleus accumbens, highlighting the reward pathway’s role in food consumption. The study concluded that “the release of dopamine by eating could be a factor in addiction to food.” Research on obesity also suggested addiction-like mechanisms, as weight regain was often viewed as a more tolerable outcome like Swanson and Dinello (1970) suggest:

*“For most patients a return to obesity was more comfortable and tolerable than trying to fight with their problem in the presence of environmental demands.”*

In the 1960s, the principles of Alcoholics Anonymous were adapted for addressing compulsive eating, resulting in the establishment of Overeaters Anonymous, followed later by Food Addicts Anonymous and Food Addicts in Recovery Anonymous in the United States (for a detailed review, see Meule et al., 2015). By the 1990s, advancements in the understanding of EDs gained momentum. In 1992, Spitzer et al. (1992) introduced the term BED, which was subsequently included in Appendix B of the Diagnostic and Statistical Manual of Mental Disorders (DSM) in 1994. During the same period, chocolate consumption emerged as a significant area of research. Bruinsma and Taren (1999) explored cravings for chocolate, examining factors such as its sensory properties, psychopharmacological effects (e.g., caffeine, theobromine, biogenic amines), self-medication theories, and hormonal fluctuations in women:

*“Chocolate cravings are often episodic and fluctuate with hormonal changes just before and during the menses, which suggests a hormonal link and confirms the assumed gender-specific nature of chocolate cravings. Chocolate contains several biologically active constituents (methylxanthines, biogenic amines, and cannabinoid-like fatty acids), all of which potentially cause abnormal behaviors and psychological sensations that parallel those of other addictive substances.”*

It is important to highlight that this theoretical foundation, along with preliminary findings, served as the basis for the more recent conceptualizations of FA in the 2000s. In 2001, a study published in *The Lancet* revealed that individuals with obesity exhibited reduced D2 dopamine receptor availability compared to their eutrophic counterparts, findings that paralleled those observed in substance addiction (Wang et al., 2001). In 2003, Kampov-Polevoy et al. (2003) reported that a family history of alcohol dependence is also associated with an increased preference for sweetness. Consequently, the concept of loss of control over food and body weight began to converge with the notion of chemical dependency. The notion of needing increasingly larger amounts of food and progressively gaining weight has long been associated with the reward deficiency theory, which is grounded in a strong neuropsychopharmacological framework. Recently, a systematic review identified 33 studies that evaluated differences in BMI between individuals with and without the A1 allele. A meta-analysis of these studies found no significant difference in BMI between carriers and non-carriers of the A1 allele (Benton and Young, 2016). These findings do not support the Reward deficiency theory of FA.

In 2009, Merlo and colleagues administered a series of questionnaires to children, including the Children’s Eating Attitudes Test (ChEAT), the Three Factor Eating Questionnaire (TFEQ), the Inventory of Overeating Situations (IOS), and the Eating Behaviors Questionnaire (EBQ). According to the authors, the EBQ includes items designed to assess the “3 Cs” of addiction—compulsive use, attempts to cut down, and continued use despite consequences. This 20-item measure was developed to evaluate hypothesized symptoms of food addiction (FA), based on adaptations of DSM-IV substance dependence criteria. The article presents a section titled “Correlates of food addiction symptoms.” Among children, the most frequently endorsed items were related to compulsive eating and a perceived lack of control (e.g., “Do you wish you could eat if you have not eaten in a while?,” “Do you want to cut down on your eating?,” and “Do you try to cut down on your eating?”). Conversely, the least commonly endorsed symptoms included items such as “Do you miss out on activities because of your eating?,” “Do you ever fight with your family, friends, or others about your eating?,” and “Do you save up or hide food?”

In 2005, Phil Werdell founded the Food Addiction Institute to address issues related to obesity and EDs (Meule et al., 2015). In 2007, Dr. Brownell and Dr. Gold initiated academic discussions on FA, which culminated in the publication of the book *Food and Addiction*. They observed that initial reactions to the concept were met with skepticism, particularly from nutrition and obesity researchers. In 2009, Gearhardt and colleagues developed the Yale Food Addiction Scale (YFAS), based on DSM-IV criteria for substance-related disorders. This was followed by the introduction of a child-specific version in 2013, and the scale was later revised in 2016 to align with DSM-5 updates. These advancements solidified the application of addiction frameworks to the study of EDs. As an alternative to FA, the term “eating addiction” was proposed (Hebebrand et al., 2014), sparking extensive debate in the literature over the distinctions and implications of these terms. Later, the Addiction-Like Eating Behavior Scale (AEBS) was developed based on qualitative data exploring self-perceived FA (Ruddock et al., 2017). The AEBQ includes items that assess individuals’ responses to food, both in terms of approach and avoidance, such as “emotional overeating” and “emotional undereating,” reflecting well-documented phenomena often altered following various treatments for obesity and eating disorders. It is important to note that many theoretical arguments in favor of FA rely on statements linking the chronicity of obesity and the recurrence of binge eating episodes to the concept of addiction. If we were to apply this logic to depression, would a relapse into depressive episodes be driven by a craving? By a loss of control over one’s mood? This leads us to the central issue of “control” in the YFAS, where individuals sometimes report difficulties in controlling themselves around food. However, the meaning of “loss of control” encompasses far more than an addictive or dependency-based relationship—it may function culturally as a label, much like the scale itself applies.

Initially, discussions around FA centered predominantly on sugar, positing it as a substance capable of eliciting addictive-like behaviors. Over time, this discourse broadened to include highly palatable foods, characterized by their combination of sugar, fat, and salt, which are thought to drive compulsive eating behaviors. More recently, attention has shifted to ultra-processed foods (UPFs), industrially engineered formulations containing minimal whole food components and relying on production techniques and additives exclusive to large-scale food manufacturing (Steele et al., 2023). This conceptual evolution reflects the growing complexity of understanding addiction-like eating behaviors within a contemporary food environment dominated by industrialized and aggressively marketed products. UPFs (Dai et al., 2024), in particular, present unique challenges, such as their hyper-palatability, low satiety index, and strategic design for overconsumption. Consequently, the narrative surrounding FA must contend with an intricate interplay of biological susceptibilities, environmental factors, and sociocultural influences, highlighting the limitations of existing theoretical models and diagnostic tools in capturing the multifaceted nature of these behaviors.

### 1.2 Measuring food addiction

Measuring psychological phenomena for psychiatry and clinical practice demands a solid theoretical foundation, rigorous testing, and validation. The YFAS was developed to address FA using diagnostic criteria adapted from the DSM-IV for substance dependence (Gearhardt et al., 2009). In the initial stages of developing the YFAS, the voices and lived experiences of individuals affected by the phenomenon were not part of the process. No qualitative studies or in-depth interviews were conducted to understand how people themselves described or made sense of what is now referred to as food addiction. Instead of beginning with participants’ perspectives, the construction of the instrument followed a more top-down approach: the item pool was drafted by the authors and then reviewed by specialists in addiction, obesity, and eating disorders (Gearhardt et al., 2009). In this review, we discuss how factors commonly associated with SUD may contribute to strong correlations with food craving, loss of control, and preoccupations with food. This may help explain why individuals diagnosed with restrictive-type anorexia nervosa (AN-r) also meet the criteria for FA+ (Granero et al., 2018) according to the YFAS. These individuals are not misreporting their cognitions or behaviors—they genuinely experience a paradoxical sense of loss of control, despite their low body weight. Nevertheless, the literature suggests that as binge eating episodes emerge within this group—specifically among those with the binge-purge subtype—the levels of FA tend to increase (Tran et al., 2020). This pattern reinforces the idea that longstanding constructs in the field of eating behavior, which are not rooted in addiction models, might sufficiently explain the so-called “FA phenomenon.” This raises a critical question: if FA were to be formally included in the DSM, how would treatment approaches for young women with AN-R and co-occurring FA+ be shaped?

The 25-item scale assesses FA related to highly processed, calorie-dense foods, focusing on criteria such as diminished control, withdrawal symptoms, unsuccessful attempts to quit, and significant impairment. It employs two scoring methods: a symptom count (0–7) and a dichotomous diagnosis based on meeting at least three criteria with clinically significant impairment. The YFAS evaluates 11 DSM-based criteria, including overconsumption, persistent desire to reduce intake, neglect of responsibilities, and withdrawal symptoms alleviated by food consumption. Its psychometric properties have been suggested as a proof of concept across diverse populations, including non-clinical groups, BED, bariatric surgery candidates, and cross-cultural contexts, supported by convergent and discriminant validity (Schulte and Gearhardt, 2017; Schulte et al., 2019; Oliveira et al., 2021). In 2016, the YFAS 2.0 was introduced to align with the DSM-5. It has 35 items scored on a Likert scale from 0 (Never) to 7 (Every day), including two items assessing distress (“My eating behavior cause me a lot of distress”) and impairment (“I had significant problems in my life because of food and eating”). It yields a total score, continuous symptom count, and categorical “diagnosis” of FA (No FA ≤ 1 symptom, and/or lack of distress or impairment; Mild FA = 2 or 3 symptoms plus distress and/or impairment; Moderate FA = 4 or 5 symptoms plus distress and/or impairment; Severe FA = 6 or more symptoms and distress and/or impairment). To improve accessibility for large-scale studies, abbreviated versions were developed. The modified YFAS 2.0 (mYFAS) maintains the DSM-5 framework into 13 items, and presents some psychometric properties, ensuring practical utility in epidemiological research.

The Addiction-like Eating Behavior Scale (AEBS) aims to quantify addiction-like eating behaviors from a perspective distinct from the clinical criteria for substance dependence. The scale is intended to identify what has been referred to as “eating addiction.” The scale captured six themes: eating for reward, persistent cravings, inability to control eating, preoccupation with food, difficulties managing weight, and struggles with high-fat, high-sugar, or high-salt foods. Analysis revealed six causal attributions for self-perceived FA and highlighted behavioral differences between individuals who identified as food addicts and those who did not. These insights informed the foundational structure of the AEBS (Ruddock et al., 2017).

### 1.3 Diagnosing FA

According to LaFata et al. (2024), although previous studies using the YFAS have contributed to understanding FA as a distinct construct and a potential indicator of more severe psychopathology among individuals with eating disorders, the lack of clinician-administered diagnostic tools has posed a significant barrier to its evaluation as a clinical syndrome. To address this gap, they developed the Food Addiction Symptom Inventory (FASI), a structured interview for the clinical assessment of FA, adapted from the substance use disorder modules of the Structured Clinical Interview for DSM-5 (SCID-5). Each FASI item maps directly onto DSM-5 criteria for SUD, yet food consumption occurs within vastly different biological, social, and psychological contexts than drug use. For instance, items assessing “overconsumption,” “persistent desire to quit,” and “tolerance” may conflate normative dietary lapses or emotional eating with compulsive pathology. Similarly, criteria such as “withdrawal symptoms” or “risky use” risk pathologizing common behaviors (e.g., eating while distracted) without sufficient empirical grounding in neurobiological addiction mechanisms. The high concordance between FA scores and symptoms of other eating disorders—such as restrictive anorexia nervosa—raises questions about the distinctiveness of FA as a diagnostic construct. These individuals are not misreporting but rather interpreting their emotional and cognitive struggles with food through the framework provided by the instrument. This suggests that FA may reflect a constellation of established constructs (e.g., craving, guilt, disordered eating attitudes) rather than a distinct disorder. Recent literature indicates that commonly cited FA symptoms, such as food craving, loss of control, and emotional distress, are often better explained by existing models in eating behavior research, including emotional regulation, dietary restraint, and cognitive preoccupation with food.

### 1.4 Are we discussing food cravings and distress caused by eating behaviors and labeling it as addiction?

Craving is a central element in the discussion of addiction. Defined as an intense and often overwhelming desire, craving has been the subject of considerable debate and theoretical exploration. Historical perspectives on craving have evolved from its initial association with physical dependence to more nuanced models that incorporate cognitive, behavioral, and neurobiological components (Skinner and Aubin, 2010; Oliveira and Cordás, 2020). Based on the comprehensive bibliographic review by Skinner and Aubin (2010), we observe that craving is a well-studied construct with numerous neural, theoretical, empirical, and neuroimaging correlates. The authors outline a series of models that integrate diverse perspectives, rooted in historical and conceptual models. Craving, as conceptualized in the literature, can be understood through various theoretical models. Conditioning-based models suggest that repeated exposure to food-related cues fosters strong associative learning, whereby specific stimuli, such as the sight or smell of food, become potent triggers for craving (Kozlowski and Wilkinson, 1987; Marlatt, 1987). Cognitive models propose that craving is perpetuated by maladaptive cognitive patterns, such as an obsessive preoccupation with food or a heightened fear of deprivation (Tiffany, 1990, 1999). Meanwhile, psychobiological models emphasize the role of interactions between brain systems involved in reward processing, emotional regulation, and stress responses, which collectively contribute to the emergence and persistence of craving (Skinner and Aubin, 2010). Also, Psychobiological models explain cravings in part by biological factors with an emphasis on motivational components. This model integrates advances in neurobiology and animal models with behavioral theories and encompasses various concepts, such as the opponent process theory, which has been applied to the case of FA as the “dark side” model (Polk et al., 2017; Parylak et al., 2011). In this framework, excessive drug use triggers the activation of the antireward system, leading to negative hedonic effects intended to reduce the experience of reward. Prolonged drug consumption disrupts neurochemical processes, resulting in an allostatic state—a chronic deviation of the regulatory system from its normal homeostatic functioning. Lastly, Motivation models suggest that craving is a component of a larger decision-making framework (Skinner and Aubin, 2010).

Furthermore, an understanding of this aspect of eating behavior can assist clinicians and patients in grasping the concepts of loss of control and difficulties in control that are associated with FA. Indeed, if patients with AN exhibit a positive FA score on the YFAS (Granero et al., 2018), it is likely that many will identify, perhaps through subjective mechanisms, the notion of being addicted to food. In these cases, it may be the case that we are dealing with (i) eating attitudes and (ii) discomfort with restrictions and FC. The study of the physiological, affective, and cognitive dimensions of various phenomena involved in seeking and selecting food represents a significant challenge in research (Skinner and Aubin, 2010). Referring to this as FA, based on criteria representing the most severe behavioral difficulties (i.e., SUD) may, ultimately, be a matter of semantics. However, it remains highly correlated with the emotional distress experienced by individuals with EDs, regardless of their degree of control. After all, individuals with restrictive-type AN may also exhibit FA. While FC are typically measured by their intensity, domains (May et al., 2015), or specific food types, none are designed to classify individuals into distinct groups. However, using a cutoff point of 50, it becomes possible to differentiate individuals with FA from those without (Meule, 2018). In contrast, the YFAS assumes difficulties in control and links these struggles to various life domains, which can often be explained by other psychological and behavioral phenomena. This approach generates a distinct conception, allowing for the classification and potential diagnosis of FA. The construct validity of YFAS relies on multiple validation studies, including evidence from psychometric properties, convergent and discriminant validity, and associations with constructs such as binge eating, obesity, and emotional dysregulation. However, challenges remain regarding the interpretation and generalization of these findings across different populations and contexts. This ongoing discussion stems from the multitude of factors influencing food-seeking behavior, contrasted with the relatively few factors that determine meal termination or the decision not to eat. Additionally, increased exposure to food-related stimuli, cultural beliefs, environmental factors, and pervasive advertising further exacerbate the brain’s stress responses related to eating behavior. Considering the various aspects involved in eating behaviors, it is relevant to analyze the different concepts, their relationships, in order to challenge the idea of FA.

### 1.5 Aims

The aim of this scoping review is to provide an overview of the studies that have investigated the relationship between FA and FC, in order to critically examine the validity of the construct of FA.

To aid comprehension, a list of abbreviations used was included for clarity (Table 1).

**Table 1:** List of abbreviations.

Definition	Abbreviation
Food addiction	FA
Food craving	FC
Body mass index	BMI
Substance use disorders	SUD
Eating disorders	EDs
Anorexia nervosa	AN
Bulimia nervosa	BN
Binge eating disorder	BED
Yale Food Addiction Scale	YFAS
The modified Yale Food Addiction Scale 2.0	mYFAS 2.0
Addiction-Like Eating Behavior Scale	AEBS
Loss of Control Over Eating Scale	LOCES
Food craving Trait and State Questionnaire	FCQ-T/S
Ultra-processed foods	UPFs
Mu-opioid receptor	MOR
Multilocus genetic profile	MLGP
Laparoscopic sleeve gastrectomy	LSG
Dorsolateral prefrontal cortex	DLPFC

## 2 Methods

### 2.1 Search strategy

This scoping review aims to broadly map the concepts and constructs related to FC and FA, taking a step back from analyzing specific outcomes to instead question and explore the methodologies employed to assess these phenomena in various contexts. A comprehensive search was conducted to identify (i) original quantitative studies involving adults, (ii) published in English, that (iii) examined FC and FA through specific methodologies, such as self-report questionnaires or visual analog scales. The systematic search was carried out up to December 2024 without time restrictions in the PubMed, Scopus, and PsychInfo databases. The search and selection process adhered to the guidelines established by the PRISMA (Preferred Reporting Items for Systematic Reviews and Meta-Analyses) framework (Page et al., 2021), adapted to the scope of this review. The search strategy employed specific terms, combining [“food addiction” or “addictive-like eating” with descriptors such as “food craving,” “desire,” “cue reactivity,” or “urge to eat.”].

### 2.2 Inclusion and exclusion criteria

Interventional or observational studies applying quantitative measures of FC (Food Craving Trait and State—FCQ-T/S or any other scales/questionnaires) and FA (any version of the YFAS or other questionnaires to measure FA were included). Studies involving children, animal models, systematic reviews, opinion articles, letters to the editor, theoretical essays, or those that did not explicitly assess FC or FA were excluded. Quantitative studies from self reported measures, surveys, experimental studies, clinical studies and randomized controlled trials were included. Longitudinal and cross sectional studies were included. Despite the existence of child-specific versions of the YFAS, the focus was intentionally limited to adolescents and adults due to the significant methodological diversity in assessing these constructs (Studies including participants aged ≥14 years).

### 2.3 Synthesis of data

Supplementary Table 2 summarizes the key instruments used to assess FC and FA across the included studies, highlighting their constructs, purposes, and corresponding references. It underscores the methodological variability in FC assessment and the consistent reliance on the YFAS for FA evaluation. The studies were summarized to include details such as the first author and year, study type, methodologies employed, analyzed diagnoses/groups, mean age and standard deviation, mean BMI and standard deviation, and measures used to assess FC/FA, along with key findings reported by the study authors (Supplementary Table 2). The presentation of results was divided into five thematic sections: 1. Genetic factors, 2. Neurobiology, 3. Behavioral factors, 4. Emotional factors, and 5. Food cues. This structure offers a comprehensive overview of the methodological strategies applied in the assessment of FC and FA, highlighting areas of convergence, divergence, and potential gaps in the existing literature. Through this approach, the review aims to contribute to a more robust and integrated understanding of these constructs. In the discussion section, we revisit the results in an integrative manner and introduce a focused critique centered on two key points: (i) theoretical and psychometric issues in FA and (ii) whether FA is fundamentally a matter of semantics. This critical reflection leads to a new understanding of the trajectory these studies have followed, shedding light on emerging perspectives and persistent gaps in the field.

## 3 Results

**Figure 1:**
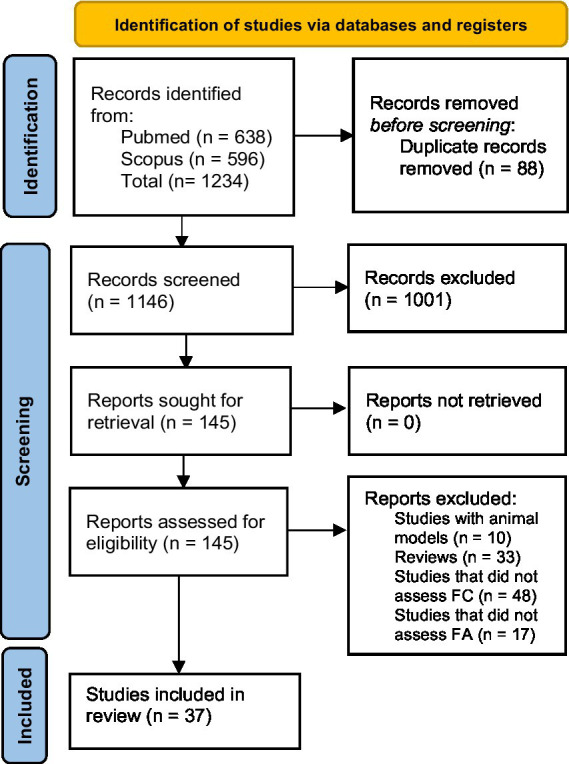
Preferred reporting items for systematic reviews and meta-analysis diagram.

Initially, 1,234 results were identified, and after removing duplicates (*n* = 88), a title/abstract analysis was performed applying the exclusion criteria. The summary of instruments utilized to assess FC and FA in the included studies were described in Supplementary Table 2. The resulting list of studies (*n* = 145) had their full articles retrieved for evaluation. Studies that did not access FC (*n* = 48) or FA (*n* = 17) were removed. At the end of the search and selection process 37 original investigations were included in this review (Figure 1; Supplementary Table 1).

### 3.1 Methods in FA and FC studies

The studies included in this review demonstrated considerable variability in the instruments used to assess FC. In contrast, FA was evaluated exclusively using the YFAS and its versions. Supplementary Table 2 presents a comprehensive overview of the key scales used in the studies reviewed, including their constructs, purposes, and references (from the present review). The studies revealed considerable heterogeneity across the populations assessed, encompassing individuals who had undergone bariatric surgery, individuals with obesity, those adhering to restrictive diets, and individuals diagnosed with EDs. This diversity in sample characteristics contributed to substantial variability in the findings related to both FC and FA. Populations exhibiting altered eating behaviors often respond distinctively to FA scale items, frequently resulting in elevated scores that classify them as meeting the criteria for FA. These elevated scores are commonly associated with heightened levels of psychological distress, including stress, anxiety, and depression, as emphasized in the synthesis of the results. This pattern raises critical concerns regarding the validity and specificity of the currently available FA assessment tools. A recurring question emerges: Does the primary FA assessment scale effectively capture a distinct and theoretically valid construct, or does it inadvertently measure overlapping phenomena related to other psychological or behavioral domains? These ambiguities call for a more nuanced understanding of the construct validity and psychometric robustness of FA assessment tools.

Supplementary Table 2 provides a comprehensive summary of the 37 studies included in this review, which examined the influence of FC and FA on EDs, cue reactivity, and treatment outcomes. It highlights their methodologies, sample characteristics, and key findings. In the following sections, we present an overview of the primary methods employed across these studies, critically analyzing their contributions and limitations.

### 3.2 Genetic factors

Researchers have shown increasing interest in studying the contributions of genetic variants, including FTO, MC4R, and LEPR, and their associations with dietary habits in recent years. In the case of FA, one theory has guided several investigations. Addiction to substances of abuse has been linked to the concept of Reward Deficiency Syndrome, which proposes that substances overstimulate reward pathways, leading to a compensatory reduction in the population of dopamine D2 receptors (Blum et al., 2014). Following the previously mentioned publication by Wang et al. (2001) and the significant impact it had on the scientific community, this model inspired a series of studies correlating YFAS scores with genetic analyses. Davis and Loxton (2014) examined individuals with obesity categorized by their alleles (GG, GA, AA) of the mu-opioid receptor (MOR), known for its role in opioid signaling and hedonic eating. Their findings demonstrated a positive correlation between carriers of the G allele and heightened hedonic responsiveness to palatable foods. MOR is activated by endogenous opioids (naturally occurring in the body, like endorphins) and exogenous opioids (such as morphine or heroin) (Krupa et al., 2024). In the context of hedonic eating. It mediates the pleasurable or rewarding aspects of eating, particularly when consuming palatable foods high in sugar or fat (Sandoval-Caballero et al., 2023; Pak et al., 2025).

Individuals with FA also exhibited higher multilocus genetic profile (MLGP) scores compared to non-FA individuals. A MLGP is a composite index that aggregates the effects of multiple functional polymorphisms within a specific neurotransmitter system to better capture individual differences in neural function. As first demonstrated by Nikolova et al. (2011), the MLGP, based on dopamine-related polymorphisms, explained a greater proportion of variance in ventral striatum reactivity than any single genetic marker considered independently, highlighting its utility in modeling biologically grounded individual differences in brain function. The MLGP score is a biologically informed polygenic index that aggregates the additive effects of multiple polymorphisms in dopamine-related genes—such as *DRD2*, *DRD4*, *DAT1*, and *COMT*—each previously linked to individual differences in striatal dopamine signaling. Rather than focusing on a single nucleotide polymorphisms (SNP), this composite score captures the cumulative influence of several functionally relevant variants across different loci, enhancing predictive power for dopaminergic activity and related behaviors. Higher MLGP scores have been positively correlated with increased binge eating, food craving, and emotional eating. These findings suggest a genetic predisposition characterized by altered dopaminergic tone, potentially contributing to compulsive eating behaviors and greater susceptibility to hyper-palatable foods (Davis et al., 2013). A meta-analysis conducted by Benton and Young (2016), examined the association between the Taq1A polymorphism (rs1800497), located near the DRD2 gene (which encodes the dopamine D2 receptor), and obesity, with implications for the Reward Deficiency Syndrome (RDS) theory. This polymorphism is linked to the density of DRD2 receptors in the striatum, with the A1 allele being associated with reduced availability of these receptors. This reduction has been interpreted, in various contexts of addiction, as a marker of decreased dopaminergic activity and a potential predisposition to addictive behaviors. However, the study concluded that a lower density of DRD2 receptors is not directly associated with increased BMI. On the other hand, among individuals who are already obese, the presence of the A1 allele may represent an additional risk factor for further weight gain, possibly through psychological mechanisms. These findings suggest that while dopaminergic genetics may influence eating behaviors, RDS alone is not sufficient to fully explain cases of FA.

### 3.3 Neurobiology

An emblematic neuroimaging study conducted 2 years after the publication of the YFAS demonstrated that FA scores are associated with increased activation in the anterior cingulate cortex, medial orbitofrontal cortex, and amygdala during the anticipation of palatable food (chocolate milkshake). Participants with higher FA scores showed greater activation in the dorsolateral prefrontal cortex and caudate during food anticipation, while exhibiting reduced activation in the lateral orbitofrontal cortex during food receipt (Gearhardt et al., 2011). Later neurobiological investigations aim to elucidate both the underpinnings of FC and the clinical responses in regions responsible for inhibitory control. Li et al. (2019) demonstrated a correlation between fasting ghrelin levels and reduced craving for high-calorie food cues, comparing Pre and Post patients who underwent laparoscopic sleeve gastrectomy (LSG) as a form of bariatric surgery, with the control group (did not receive LSG surgery). Their results revealed decreased activation in the right dorsolateral prefrontal cortex (DLPFC) under high-calorie versus low-calorie food cues, along with increased connectivity between the right DLPFC and the ventral anterior cingulate cortex. These findings suggest that weight loss induced by LSG may reduce FC by modulating hormonal levels and neurocircuitry responses. Further examining prefrontal cortex functions, Leong et al. (2018) investigated the role of infraslow neurofeedback targeting the posterior cingulate cortex in individuals with obesity and FA symptoms. Their findings indicated that neurofeedback sessions altered brain activity and reduced FC. Ho and Verdejo-Garcia (2021) suggest that biopsychosocial models that integrate food, neurobiology and context can provide a better understanding of compulsive eating manifestations in a transdiagnostic framework. Considering these results, it’s important to highlight that neuroimaging studies in the field of SUD typically rely on a combination of diagnostic interviews, structured assessments, and self-report questionnaires to measure phenomena with psycho neurobiological correlates. In the case of FA, it is important to emphasize the central role of the YFAS—a self-report tool—in assigning individuals to FA groups. This reliance raises concerns, as the correlations established between FA symptoms and neural activity are, in effect, driven by the scale itself, potentially reinforcing circular validation. For instance, in studies of depression and neuroimaging, would it be methodologically acceptable to base group classifications solely on self-report measures and then claim the scale’s validity based on the resulting correlations? This approach raises critical questions about construct validity. Food-related phenomena such as preoccupation with eating, guilt, craving, compulsive behavior, and heightened reactivity to food stimuli have identifiable neural correlates (Meye and Adan, 2014). But do these findings explain FA specifically—or are they better understood as features of broader disordered eating behaviors? In this context, it is necessary to question whether the observed neural patterns reflect a distinct addiction-like syndrome, or whether they simply mirror the complexity of eating pathology without necessitating an addiction framework.

### 3.4 Behavioral factors

The investigation of eating behaviors and their underlying mechanisms has emerged as a pivotal focus of research, especially within the realms of EDs and FA. Patterns related to eating behaviors and cravings have been widely studied. Meule et al. (2014a) reported that individuals with FA exhibited elevated cravings, ED-related psychopathology, and depressive symptoms before bariatric surgery, alongside associations with impulsivity and binge eating frequency. Post-bariatric surgery, Pepino et al. (2014) observed significant improvements in eating behaviors, with remission of FA symptoms and reductions in emotional and external eating, supporting better weight management outcomes. Giel et al. (2017) examined inhibitory control in women with BED, reporting improved responses and fewer binge episodes after a cognitive intervention. Interventions focusing on food-related impulsivity appear to be promising, particularly concerning binge eating frequency, and also for FC and inhibitory control (İnce et al., 2021). Meule et al. (2012) showed that heart rate variability (HRV) biofeedback training effectively reduced EDs compared to a control group. Biofeedback training is a therapeutic technique that involves teaching individuals to regulate their physiological responses by improving the balance between the sympathetic and parasympathetic nervous systems, promoting stress management. Using a stop-signal task, a behavioral paradigm to measure response inhibition, Meule et al. (2014b) found that delayed responses to food cues were linked to heightened cravings, while Meule and Kübler (2012) identified perceived lack of control, intentions to eat, and triggers from food cues as significant predictors of FA symptoms. The relationship between these FC subscales reveals various motives for eating tied to desire and consumption, and they appear to closely mirror the criteria for craving proposed in FA. Similarly, Wiedemann et al. (2022) categorized participants by FA severity and found a strong association between higher FA severity and increased symptoms of EDs, loss of control over eating, and cravings. Research on bariatric surgery has provided additional insights into eating behavior dynamics. The Loss of Control over Eating Scale (LOCES) is a psychometric tool that offers valuable insights and evidence regarding the interplay of various constructs associated with the concept of FA. The LOCES evaluates the emotional and psychological experience of losing control during eating episodes, capturing feelings such as an inability to stop eating, preoccupation with food, and a sense of powerlessness during these episodes (Latner et al., 2014). A greater sense of loss of control was independently and more strongly associated with eating psychopathology, dissatisfaction with body shape, hunger, FC, and symptoms of FA. In contrast, larger binge sizes were more closely tied to concerns about weight and reduced general and social quality of life. Both loss of control and binge size were linked to eating-related concerns but showed no significant association with depression or BMI. These findings emphasize the importance of addressing loss of control and binge size as primary psychosocial treatment targets for individuals with BED (Bruzas et al., 2022). Gaining insights into the intricate interplay between cravings, emotional regulation, and behavioral patterns is urgent for unraveling the complexities of EDs and advancing the development of effective, targeted interventions.

### 3.5 Emotional factors

Emotional factors play a key role in understanding FC and its relationship with ED reinforcing the notion that individuals classified with FA exhibit greater severity regarding emotional comorbidities and difficulties, which manifest in EDs. Davis et al. (2011) found that individuals with FA symptoms exhibited higher rates of BED, depression, and attention-deficit/hyperactivity disorder (ADHD) compared to those without FA symptoms. Furthermore, the FA group reported elevated FC, binge eating episodes, and emotionally driven eating. El Archi et al. (2020) investigated FC intensity in individuals at risk for AN, BN, and BED, showing higher FC intensity in individuals at risk for BED and BN compared to AN, with BN participants exhibiting the highest FA prevalence. Joyner et al. (2015) explored FC’s mediating role in the relationship between FA symptoms, BMI, and binge eating episodes. Their results suggested that FC partially mediated these associations, linking FC with overconsumption and BMI elevation. Similarly, Meule et al. (2017) evaluated the psychometric properties of YFAS 2.0, finding that higher scores were associated with greater eating pathology and attentional impulsivity across populations, including bariatric patients. Wang and Lopez-Fernandez (2019) conducted a web-based survey exploring FA correlates, revealing that individuals with three or more FA symptoms were more likely to be female, have higher BMI, and exhibit lower self-esteem and higher narcissism levels. Studies show a high prevalence of comorbidities among individuals exhibiting EDs behaviors. Moreover, high FC or FA scores are more prevalent in individuals with AN, BN, or BED, with particular emphasis on BN and/or BED.

### 3.6 Food cues

Research on food cues highlights their impact of FC on FA dynamics. Cue-reactivity research initially focused on classical conditioning as the primary explanation for cravings. However, it is now understood that cravings are shaped by a complex interplay of conditioning, cognitive processes, and biological mechanisms. The modern food environment further complicates this issue, with marketing strategies designed to intensify cravings and encourage the excessive consumption of highly palatable foods (Steele et al., 2023). Schulte et al. (2017) reported that UPFs elicited stronger addictive-like responses than minimally processed alternatives, emphasizing their abuse liability. Strategies exploit pleasure-seeking behaviors, often leading to heightened cravings and a reliance on food for emotional regulation, especially during periods of negative affect or dysphoria. The loss of control over eating is further amplified by ineffective weight-loss strategies and the persistent difficulty of maintaining healthy dietary habits in contemporary society. Research by Wiedemann et al. (2021) demonstrated significantly higher levels of dietary restraint among individuals classified within mild or severe FA groups. Similarly, Chao et al. (2019) identified elevated FC in individuals with obesity meeting FA criteria, noting a reduction in these cravings following behavioral weight-loss interventions. Moreover, avoidant thoughts about food, concerns over weight gain, and dissatisfaction with body image exacerbate emotional dysregulation and complicate decision-making around food choices, as highlighted in studies by Schulte and Gearhardt (2017), Schulte et al. (2019), and Yates et al. (2024). Similarly, Davis et al. (2014) found that individuals with FA exhibited heightened FC and appetite ratings in response to methylphenidate compared to non-FA participants. Gearhardt et al. (2014) analyzed craving responses to high-fat foods, finding a moderate association between fat content and FC. Polk et al. (2017) noted that cognitive restraint negatively predicted cravings for UPF, while FA symptoms positively predicted craving intensity. These findings were corroborated by Schulte et al. (2019), who observed a robust association between UPF and indicators of addictive behaviors. These interconnected factors underscore the complexity of addressing FA and related eating behaviors.

## 4 Discussion

FA has gained significant attention in food science and nutrition. Its clinical implications and applicability remain a subject of debate. Before describing the correlations between FC and FA presented in this review, we aim to focus on the quality and type of measures used, emphasizing the diversity and complexity of methodologies employed to assess FC in contrast to the single measure used to evaluate FA—YFAS and its versions. It is evident that the conceptual, theoretical, psychometric, and practical challenges associated with the use of the YFAS—including its symptom count, the clarification of FA prevalence (%) in samples, and the interpretation of correlation results—must be critically considered. Does merely developing a scale with questions about how food might alleviate emotions validate the concept of emotional eating? Scales do not create psychological constructs. Even if they demonstrate high correlations and discriminative power in relation to related constructs, this does not necessarily mean they measure what they are intended to measure. Also, the lack of evidence supporting the notion that sugar or specific foods, like UPF cause FA does not automatically imply the existence of eating addiction. It is urgent that these concepts are rigorously investigated and demonstrate theoretical and epistemological coherence, as they are increasingly being used as outcomes in interventions and treatments, as will be discussed in this review.

The structure of the YFAS survey inherently guides respondents toward reflecting on difficulties in [controlling food intake]. As stated explicitly, *“This research asks about your eating habits over the past year. People sometimes have difficulty controlling how much they eat of certain foods, such as [….] When the following questions ask about ‘Certain foods’, please think of any foods or drinks similar to those listed in the groups above or any other foods you had difficulty controlling your consumption of over the past year.”* By framing the questions in this manner, the survey presupposes the existence of a struggle with control, which may not be exclusive to individuals with elevated BMI, binge eating, or obesity (Schulte and Gearhardt, 2017). When a respondent is presented with a stimulus, such as a series of test items, their responses are interpreted as reflections of an underlying latent trait (Pasquali, 2009; Zanon et al., 2016). The assumption is that these responses correlate with the intensity or presence of the said trait. However, in this case, the framing primes respondents to interpret their behavior through the lens of “difficulty in control,” potentially leading individuals across diverse categories—those with low BMI (Schulte and Gearhardt, 2017), individuals with restrictive AN (Granero et al., 2018; Tran et al., 2020), those without any ED, and even those with ED or obesity—to identify with the construct of FA at some level. This raises a critical question: if everyone can exhibit some degree of FA based on these criteria, what does this “construct” truly represent? Does it meaningfully differentiate between pathological and non-pathological eating behaviors, or does it instead reflect a universal human experience shaped by external stimuli and subjective interpretation? Could the distraction caused by food and its effects be comparable to substance use, to the extent that a person might have been harmed, as in the question: *“#12 I was so distracted by eating that I could have been harmed (e.g., while driving a car, crossing the street, operating machinery)”?* Eating behavior can cause significant distress that extends beyond the parameters of EDs and weight-related concerns, as reflected in question *#5: “My eating behavior has caused me significant distress.”* Fear of weight gain, body dissatisfaction, cravings, failed dieting attempts, a history of weight-related stigma, frequent and intense emotional eating, among other factors, all intersect with the notion of loss of control. But does this notion of loss of control truly correspond to FA? Even when analyzing control for ED symptoms assessed through various instruments, the semantic and subjective attribution remains. An instrument is considered valid if it accurately measures what it is intended to measure. An instrument cannot be deemed fully valid if it captures items associated with another diagnosis or an entirely different clinical, neurological, and behavioral category, such as SUD. While extensive research has explored these constructs independently, this review sought to clarify their associations and implications, particularly concerning obesity, overweight, and EDs. In the following sections, we critically examine the implications of using the YFAS in these studies, the correlations identified, and the directions this body of literature has suggested.

### 4.1 Biological and behavioral factors

Under biological factors, we consider genetics and neurobiological aspects. Studies examining genetic and neurobiological factors have provided valuable insights into the mechanisms underlying FC and its potential link to FA. Alterations in regions such as the prefrontal cortex, amygdala, hippocampus, and nucleus accumbens disrupt the integration of motivational and learned information, resulting in inappropriate behavioral responses. Neurobiological theories emphasize dopamine’s role in assigning motivational value to drug-related cues. Neuroimaging studies have shown heightened activation of the brain’s reward circuitry in response to drug-related stimuli, underscoring the importance of craving as a key phenotype in addiction research. Functional connectivity between different brain networks suggest that disruptions in reward-related brain networks may contribute to addictive eating behaviors (Ravichandran et al., 2021). The dopamine receptor 4 (DRD4) in the prefrontal cortex mediates key aspects of behaviors such as cognitive control and motivation, and has been implicated in behavioral inhibition and responsiveness to food-related cues. Building on this framework, the study by Portella et al. (2021) investigated how DRD4 gene expression in the PFC influences brain responses to food images in adolescents. Their findings revealed that, among girls, lower predicted DRD4 expression was associated with reduced activation in brain regions involved in attention, cognitive control, and reward evaluation when viewing images of palatable foods (Portella et al., 2021). The issue with these findings lies in the fact that established methodologies and diagnostic frameworks already encompass various domains of eating behavior, such as FC, emotional eating, overeating, and, in some cases, binge eating behaviors commonly observed in EDs and disordered eating. Consequently, FA appears to intersect across all these domains, functioning both as a marker of severity and as a proposed distinct diagnostic category.

Genetic variations associated with enhanced dopamine signaling have been implicated in heightened responsiveness to palatable foods, suggesting a potential predisposition to addictive-like eating behaviors (Davis et al., 2013). Furthermore, dysfunctions in brain systems involved in motivated behavior, memory, stress reactivity, decision-making, and executive control play a central role in addiction. Moreover, the MLGP score, indicative of genetic susceptibility, was found to be higher in individuals who scored higher for FA and positively correlated with binge eating, emotional eating, and FC (Davis et al., 2013; Meule et al., 2014b). It has been postulated that drugs stimulate the brain’s reward mechanisms so intensely that, to compensate, the population of dopamine D2 receptors (DD2R) declines, a phenomenon reflecting a ‘Reward Deficiency Syndrome.’ As a result, increased consumption is necessary to achieve the same level of reward, while the absence of additional intake leads to cravings and withdrawal symptoms. It has similarly been suggested that FA, like drug abuse, may contribute to a reduction in DD2R density. Consequently, the role of DD2R in obesity was examined by investigating the association between BMI and the Taq1A polymorphism, given that the A1 allele is associated with a 30–40% reduction in the number of DD2Rs. A systematic review by Benton and Young (2016) identified 33 studies evaluating BMI differences between individuals with and without the A1 allele. A meta-analysis of these studies found no significant difference in BMI between carriers and non-carriers of the A1 allele. These findings do not support the Reward Deficiency Syndrome as an explanation for FA.

According to Schur and Carnell (2017), current research findings on single-gene polymorphisms have not yet converged to identify specific biological pathways or affected brain regions. They emphasize that studies employing whole-brain analytic methods, incorporating both lean and obese individuals carrying risk alleles, and rigorously controlling for satiety state, may lead to more consistent and reliable outcomes. Moreover, given the substantial heritability of obesity and the likelihood that these genetic influences manifest at the level of brain function, it is crucial to account for potential genetic confounding in obesity research. Schur and Carnell (2017) caution against interpreting obesity or excessive adiposity as solely acquired traits associated with distinct fMRI response patterns, suggesting that such interpretations may underestimate the significant roles of inherited susceptibilities, as well as behavioral and environmental factors, in shaping brain responses to food cues.

If on one hand it is a collective erroneous assumption that people living with obesity and overweight would be more prompt to develop FA, on the other hand there are individuals with anorexia also scoring FA+. Notably, the assumption of FA in individuals with AN presents a unique challenge, highlighting the complexity of these conditions and the need for further exploration of their underlying mechanisms (Granero et al., 2018). Mallorquí-Bagué et al. (2020) describe that AN exhibited greater FA, greater use of maladaptive strategies, and emotional dysregulation. While studies have demonstrated associations between hedonic responses and neural activity, relying solely on the YFAS is insufficient to validate FA as a distinct construct. All well-established constructs of ED psychopathology already have known neural correlates and patterns associated with EDs, obesity. Future studies in EDs and eating behaviors should address the ambiguities introduced by the YFAS in relation to these pre existing constructs.

The complex interplay between FC and the labeling of such experiences as “addiction” involves multiple behavioral, cognitive, and cultural factors. For instance, imagining the taste of a cookie and anticipating the pleasure of consumption can trigger cravings regardless of physiological hunger (May et al., 2015). Similarly, cravings can extend to substances like caffeine or even essential elements like water during dehydration (Kavanagh et al., 2005). One significant contributor is the level of food processing, which has been shown to influence FC (Gearhardt et al., 2014). However, beyond the palatability of food, cultural learning and food representation also play a role in shaping psychological responses to eating. How individuals perceive food—as a source of pleasure or a contributor to weight gain—profoundly affects their emotional and cognitive evaluations (Gearhardt et al., 2014). The incentive sensitization theory focuses on neuroadaptations in the brain’s reward system, leading to heightened sensitivity to drugs or drug-related stimuli. In this model, craving is described as “wanting,” distinct from “liking,” reflecting the neural assignment of motivational salience. This assignment plays a role in driving compulsive and maladaptive drug-seeking behaviors (Robinson and Berridge, 2024).

Behavioral factors, including cognitive processes and response inhibition, have been extensively studied in relation to FC and eating behavior. Research suggests that individuals with higher levels of cognitive restraint may exhibit increased FC and reduced success in self-regulating weight (Meule, 2017). Furthermore, interventions such as response inhibition training and HRV biofeedback have shown promise in mitigating symptoms associated with EDs (Meule et al., 2012; Giel et al., 2017). The correlation between cognitive and behavioral processes highlights the intricate nature of FC and underscores the potential utility of cognitive interventions in addressing compulsive eating behaviors. If these treatments reflect lower levels of FC and FA in follow-up studies, does this mean we are treating addictions? Or are we addressing complex cognitive functions that exhibit known dysregulations and associations (e.g., emotional eating, impulsivity, binge eating, and eating concerns)? Understanding subjective dynamics without rushing to classification presents a significant challenge in both clinical and research settings, where categorization often seems mandatory.

Exploring the spectrum that encompasses (i) cravings, (ii) EDs, and (iii) distress related to body image is a complex task for researchers, particularly if they fail to grasp the human narratives underlying this multifaceted phenomenon. For example, all FC subscales except for [anticipation of positive reinforcement] positively predicted FA symptoms while positive reinforcement negatively predicted FA symptoms (Meule and Kübler, 2012). This again suggests that FA functions either as a marker of severity or as a scale that assesses (i) beliefs and attitudes toward eating and (ii) distress related to one’s relationship with food, particularly concerning issues of control. Such societal pressures often intersect with personal thoughts, emotions, and cultural norms, shaping both food avoidance and food-seeking behaviors. These sensations coexist within individuals’ minds alongside societal stigmas surrounding weight, body image, and the relentless pursuit of aesthetic ideals.

### 4.2 Theoretical and psychometric issues in FA

An analysis of the theoretical and methodological construction of the YFAS can benefit from a comparison with established principles for psychometric instrument development, such as those outlined by Pasquali (1999) and organizations like the International Test Commission (2001) and the American Educational Research Association, American Psychological Association, and National Council on Measurement in Education (1999) (Reppold et al., 2014; American Psychological Association, n.d.). The process of constructing instruments should follow three primary axes: theoretical, empirical, and analytical procedures, which ensure the instrument’s validity and reliability. While the YFAS has received psychometric validation across diverse populations (Oliveira et al., 2020), and its structure has been widely supported, a more detailed critique using these guidelines reveals potential areas for improvement. The DSM criteria, originally designed to address chemical substance dependencies, may not fully encapsulate the multifaceted nature of eating behaviors. Pasquali (1999) emphasizes the importance of accurately specifying the behavioral categories that define the construct to be measured. In this context, the YFAS could benefit from broader theoretical considerations of the psychological and sociocultural complexities underlying food-related behaviors, which the DSM framework might not adequately capture. During the development of the YFAS, no qualitative studies or in-depth interviews were found that captured individuals’ symptoms, experiences, or descriptions of what FA entails. On the contrary, such studies only began to emerge after the instrument had already been developed and validated, as noted by several authors. Throughout the development process of the YFAS, individuals were not interviewed about the phenomenon; instead, the procedure followed was as described below:

“*The original pool of items was developed by the authors prior to review by experts in the addiction, obesity and eating pathology fields. The experts were asked to review item content and question wording and to indicate any criteria that they believed were not adequately assessed*” (Gearhardt et al., 2009).

Despite the effort, the authors criticize the use of self-report during the scale development process in the following excerpts:

“*One study found that “chocolate addicts” had physical, behavioral, and emotional responses to chocolate that were similar to drug addicts’ responses to drug cues* (Tuomisto et al., 1997)*. Although, these findings are intriguing, self- identification was used to classify the group of “chocolate addicts.” Self-identification may be especially problematic, as those who are dependent often lack insight into the existence or extent of their problems* (Farid et al., 1998).”

Gearhardt et al. (2009) emphasize that the YFAS was designed to identify signs of addiction to specific types of food. Their approach was based on the similarity between SUD and dysfunctional eating behaviors. In other words, the YFAS is not grounded in the construct of food addiction itself, as, at the time, there were no validated instruments developed in accordance with a well-defined conceptualization of this construct. The operationalization of the YFAS remains based on self-report, through items developed and validated by experts that capture concepts from SUD, with clear psycho neuropharmacological correlates. However, what is the relevance of this framework to individuals’ actual eating behavior? The operationalization of constructs into items, another key component of theoretical procedures, also warrants scrutiny.

When we consider the example of the development of the AEBS, a different methodological approach becomes evident. In this case, six identified themes reflect constructs that were already well-established in the field of eating behavior: (1) eating for reward, (2) persistent cravings, (2) inability to control eating, (3) preoccupation with food, (4) difficulties managing weight, and (5) struggles with high-fat, high-sugar, or high-salt foods (Ruddock et al., 2017). Impulsivity, compulsivity, cognitive flexibility, emotional eating, and eating attitudes (i.e., thoughts, beliefs, and feelings about food) clearly have neural correlates that have been extensively explored in neuroscience. These correlates appear to be sufficient to explain the difficulties individuals face in regulating their eating behavior, as well as the dilemmas posed by an obesogenic environment and the aggressive marketing of ultra-processed foods and consumption-related stimuli.

The YFAS appears to rely heavily on DSM criteria, which might limit the incorporation of insights derived from qualitative studies. Pasquali (1999) emphasizes that validation extends beyond statistical analysis. Moreover, the reliance on statistical outputs does not inherently confirm that the instrument measures the intended construct with precision (Reppold et al., 2014). It is important to consider that semantics can uncover statistical correlations between terms related to eating behavior and constructs that are already known and validated. In this sense, it becomes possible to develop an addiction scale for virtually any behavior that causes harm or that individuals attempt to control, by applying the diagnostic criteria of SUD. This may lead to (i) forced correlations derived from phenomena already known in the context of SUD, and (ii) redundancy between terms due to linguistic overlap and subjective experience—for instance, between ‘difficulties in control’ and ‘intentions to control eating,’ or ‘concerns about food and body.’ Cravings emerge as a major indicator of such redundancy. Future studies could investigate how each of the well-established constructs in the field of eating behavior contributes to explaining the FA factor. If these constructs fully account for FA, then the YFAS-based definition of FA may represent a constellation of already known behavioral constructs rather than a distinct diagnostic entity. These hypotheses should be examined in depth in future research.

While the adaptation of SUD frameworks offers structural clarity, we argue that this approach risks conceptual misalignment when applied to eating behaviors. Below, we offer a critical review of each diagnostic item included in the FASI, underscoring theoretical, psychometric, and ecological issues. Based on the literature reviewed, the studies addressing FC and FA in this analysis, and the extensive content explored throughout the development of this work, we present a set of hypotheses and proposals to be tested using the FASI in future research.

#### 4.2.1 Overconsumption beyond intent

*Criterion: Consumption of UPF in larger amounts or over longer periods than intended.*

This item assumes that exceeding dietary intentions represents a loss of control indicative of addiction. However, such behavior is widely observed in normative populations under conditions of dietary restraint, emotional distress, or environmental cue exposure. Do conditioning and cognitive models sufficiently explain such responses without requiring an addiction framework, thereby making this criterion vulnerable to false positives?

#### 4.2.2 Unsuccessful attempts to cut down

*Criterion: Persistent desire or unsuccessful efforts to reduce or control UPF consumption.*

This criterion does not distinguish between pathological compulsivity and normative dieting behavior, which is widespread and culturally reinforced. The literature shows that failed dietary control is common, particularly among individuals experiencing weight stigma or restrictive norms, without implying neurobiological addiction mechanisms. Might this criterion reflect cultural pressures rather than an underlying psychopathology?

#### 4.2.3 Excessive time involvement

*Criterion: Considerable time spent obtaining, using, or recovering from UPF.*

Time spent thinking about or consuming UPF can be confounded with food-related preoccupation due to chronic dieting, body dissatisfaction, or food insecurity. Does the assumption that time investment equates to compulsivity accurately reflect the psychosocial context in which food is prioritized, particularly in emotionally regulated eating patterns?

#### 4.2.4 Craving

*Criterion: Intense desire or urge to consume UPF.*

Craving is a complex and multifaceted construct with established theoretical models (e.g., conditioning, cognitive, psychobiological). Does using it as a standalone addiction criterion risk conflating a common human experience—often intensified by restriction, stress, or emotional triggers—with compulsive drug-seeking behavior? This item overlaps with constructs such as emotional eating, diminishing its discriminant validity.

#### 4.2.5 Functional impairments

*Criterion: Failure to fulfill major obligations due to UPF consumption.*

Attributing occupational or academic underperformance to UPF consumption may conflate correlation with causation. Such impairment may instead stem from comorbid mental health conditions, stress, or socioeconomic factors. Could self-reported impairment reflect internalized guilt or stigma surrounding eating, rather than behavior driven by a compulsive need?

#### 4.2.6 Social/interpersonal problems

*Criterion: Continued UPF consumption despite interpersonal conflicts.*

Conflicts over eating may arise from family expectations, aesthetic pressures, or moral discourses about food. This criterion risks medicalizing sociocultural tensions, and fails to consider the possibility that social conflicts might not stem from true behavioral dysregulation but from external judgment.

#### 4.2.7 Abandonment of activities

*Criterion: Reduced or abandoned activities due to UPF consumption.*

While this is a key criterion in SUDs, its application to food behavior lacks nuance. Withdrawal from social or leisure activities may result from comorbid depression or anxiety, and the association with UPF may be indirect. Without controlling for these variables, the criterion risks over-attribution.

#### 4.2.8 Risky situations

*Criterion: Recurrent UPF consumption in physically hazardous contexts.*

Eating while driving or during other tasks is common and not inherently risky. The adaptation of this item from SUD lacks ecological validity when applied to food. There is insufficient evidence that UPF consumption in such contexts entails comparable harm or loss of awareness seen in substance use.

#### 4.2.9 Continuation despite harm

*Criterion: Continued UPF use despite knowledge of physical or psychological harm.*

The chronic nature of health conditions such as obesity or diabetes complicates this item. Continuation of eating behavior despite these conditions may stem from emotional regulation needs, not addictive drive.

#### 4.2.10 Tolerance

*Criterion: Need for increased UPF amounts to achieve effect or reduced effect with same amount.*

Tolerance is difficult to operationalize in eating. Could increased intake be better explained by hedonic habituation, stress coping mechanisms, or cultural eating norms rather than by a neuroadaptive process? The lack of neurobiological markers to validate food-related tolerance further weakens the criterion’s applicability.

#### 4.2.11 Withdrawal

*Criterion: Physical or psychological symptoms upon reduction or cessation of UPF.*

Symptoms such as irritability or anxiety may emerge due to psychological deprivation or unmet emotional needs, not physiological withdrawal. Does this criterion risk pathologizing emotional eating or the common discomfort associated with restriction, particularly in contexts where food is morally coded or tightly regulated?

Across all items, the instrument closely mirrors DSM-based SUD diagnostic criteria but lacks grounding in lived experience and in theoretical models specific to eating behavior. As highlighted in the critique, several items overlap with well-established constructs such as emotional eating, dietary restraint, and binge eating, raising concerns about construct redundancy. In addition, semantic overlap between “addiction-like” and culturally shaped responses—such as guilt, desire, and social judgment—may distort the interpretation of results. This issue is further complicated by prevalence data. Studies indicate that FA is more frequently comorbid with BED than with other eating disorders (OR = 1.33, 95% CI: 0.64–2.76; *χ*^2^ = 4.42; *p* = 0.44; *I*^2^ = 0%), though this difference is not statistically significant. When compared individually, FA shows higher odds in patients with AN, restrictive type (OR = 8.75, 95% CI: 1.08–70.70; *p* = 0.04), and binge/purging type (OR = 1.93, 95% CI: 0.20–18.92; *p* = 0.57), as well as in individuals with obesity (OR = 5.72, 95% CI: 3.25–10.09; *p* < 0.0001), and in the general population (OR = 55.41, 95% CI: 8.16–376.10; *χ*^2^ = 18.50; *p* < 0.0001; *I*^2^ = 0%). Interestingly, FA appears less prevalent among individuals with BN (OR = 0.85, 95% CI: 0.33–2.22; *χ*^2^ = 0.35; *p* = 0.74; *I*^2^ = 0%) (di Giacomo et al., 2022).

Notably, the presence of FA in individuals without diagnosed mental illness—and even within the general population—raises further questions about whether FA represents a distinct diagnostic entity or a set of overlapping behavioral and emotional constructs. For example, the detection of FA in individuals with restrictive-type AN, who by definition exert control over intake, underscores the possibility that the instrument may be capturing emotional and cognitive distress. These individuals are not misreporting, but interpreting their struggles through the framework imposed by the instrument.

### 4.3 FA is a matter of linguistic-cultural components?

The studies analyzed in this study corroborate the relationship between psychosocial distress, eating behavior issues, and FC, highlighting how these interconnected factors contribute to a perceived sense of addiction or difficulty with the feeling of loss of control. Individuals who report addictive-like eating behaviors frequently describe multiple dimensions of FC, including intentions to consume food, anticipation of relief from negative emotional states, and a lack of control over eating episodes (Meule and Kübler, 2012; Chao et al., 2019). Importantly, FC is not solely determined by the properties of the desired food, but it is also shaped by attitudes and perceptions surrounding cravings. Cultural learning, food representation, and the moralization of food—particularly in relation to its level of processing—play a subjective role in shaping eating behaviors and responses to FC (Eller, 2014; Mayes and Thompson, 2015). While some cravings may be driven by fundamental survival instincts, others are heavily influenced by societal perceptions of specific foods as “fattening” or “addictive.” It is plausible that individuals use the term “addiction” synonymously with desire, fear of loss of control, and associated feelings of guilt or failure. These sentiments align closely with criteria endorsed in the YFAS. In addition, it has been observed that FA and addictive-like behaviors have been presented as synonyms. Furthermore, the widespread reliance on self-report measures, such as YFAS, highlights the inherently subjective nature of these constructs. These findings suggest that FC and the perception of FA cannot be fully understood without considering the complex interplay of emotional, cultural, and cognitive factors. Navigating self-control in an obesogenic environment further complicates the relationship between FC and FA. The constant exposure to highly palatable, ultra processed foods challenges individuals’ ability to regulate their food intake effectively. In such contexts, self-control emerges as a critical mechanism for managing cravings and resisting the temptations posed by the environment. The abundance and accessibility of hyper-palatable foods, combined with marketing strategies that emphasize immediate gratification, create a setting where self-control is persistently tested. This dynamic reinforces the subjective perception of “addiction” and complicates efforts to distinguish between loss of control due to biological drives and patterns reinforced by environmental cues.

### 4.4 Conclusion

The concept of FA, along with its associated scales and the recently developed clinician-administered diagnostic questionnaire, integrates cultural perceptions of food with established psychological constructs, drawing on previously recognized phenomena. Investigating the continuum that encompasses (i) FC, (ii) disordered eating attitudes, and (iii) body image–related distress presents a significant challenge—particularly when researchers overlook the human narratives and sociocultural dynamics that define this multifaceted phenomenon. However, the inability to achieve universal success in weight loss or to fully eliminate obesity and EDs does not necessarily imply that individuals are addicted to ultra-processed foods UPF or any other type of food. This explanatory framework risks oversimplifying the complex behavioral, emotional, and environmental factors involved in eating behavior. Therefore, it is essential to critically assess the theoretical and empirical foundations of FA before endorsing it as a definitive clinical construct or outcome measure.

While neurobiological studies—such as those evaluating changes in appetitive hormones and neural activity following bariatric surgery—have contributed to our understanding of food motivation, there remains a need to investigate behavioral strategies aimed at reducing FC, particularly in preoperative contexts. In this light, FC should be understood as a phenomenon influenced by biological, psychological, and environmental dimensions. Although it remains uncertain whether food itself possesses addictive potential in a pharmacological sense, the subjective experience of craving—especially when coupled with perceived loss of control—generates a compelling narrative that resembles addiction. Understanding the complexities underlying this experience is essential for developing more precise and personalized interventions to address problematic eating and to foster healthier relationships with food.

Collectively, the present findings support the view that FA reflects a cluster of preexisting psychological constructs—such as craving, guilt, and disordered eating attitudes—rather than a clearly delineated disorder. These findings underscore the importance of adopting a comprehensive approach to understanding and treating what is being referred to as “addictive eating behaviors,” considering the diverse perspectives from which these phenomena emerge. Future research can better clarify the mechanisms underpinning FC and FA, paving the way for more effective clinical and public health interventions focusing on improving the wellbeing of the population.
