# Supplementary material for: Questioning the validity of food addiction: a critical review
Source: Front Behav Neurosci. 2025 Jul 30;19:1562185. doi: 10.3389/fnbeh.2025.1562185 (PMC12343691; doi:10.3389/fnbeh.2025.1562185)
Supplement: Supplementary file 1 [file Table_1.docx]

**Supplementary Table 1. Studies which investigated the influence of food cravings and food addiction in disordered eating, cue-reactivity and treatment outcomes (n=37)**

| **Author, year** | **Type; Aims/Methods** | **Participants** | **Age** ± **SD (years)** | **BMI**  ± **SD (kg/m^2^)** | **Food Craving (FC)** | **Food Addiction (FA)** | **Main results** |
| --- | --- | --- | --- | --- | --- | --- | --- |
| **General Labeled Magnitude Scales (gLMS)** | | | | | | | |
| Polk, 2017 | Cross-sectional study;  FC and liking were evaluated for 35 nutritionally diverse foods, categorized as processed if they contained added fat and/or refined carbohydrates. Of these, 18 foods were highly processed, and 17 were minimally processed. Participants rated their liking for each food on a scale ranging from -100 ("Greatest dislike") to 100 ("Greatest like"). | n= 216  61.6% were female (n= 133) | 38.15 ± 14.02 | 27.98 ± 6.28 | Average FC of 37.33, on a scale from 0 (“No craving at all”) to 100 (“Strongest  craving every experience of any kind”). | Symptoms:  1.93 ± 1.56 | FC was notably higher for highly processed foods, and this association strengthened with an increased YFAS symptom count. In contrast, the link between liking and food processing was weaker. Participants with higher cognitive restraint reported lower FC and liking for highly processed foods. Interestingly, among individuals with elevated BMI, FC for highly processed foods remained stable, while liking increased. Importantly, the findings also distinguished between FC and liking, showing that YFAS indicators of food addiction were positively associated with FC, but not with liking, for highly processed foods. |
| **Visual Analogue Scales (VAS)** | | | | | | | |
| Gearhardt, 2014 | Experimental study;  Over a two-hour session, participants completed self-reported measures of current hunger, a food-related visual-search task, FC and liking ratings for various foods, and self-reported assessments of eating pathology and behaviors. FC and liking ratings were collected for 180 food images varying in sugar, fat, and processing levels. | Women with overweight and obesity (n= 89). | 31.27 ± 9.70 | 35.07 ± 8.05 | 2.97 (seven-point Likert rating) | Symptom count: 3.06 ± 2.13. | In this study, women with the highest BMIs reported lower craving magnitudes for fattier foods. While higher fat content was moderately linked to greater FC, attempted dietary restraint showed no association with either FC or food liking. Additionally, increased state hunger was associated with elevated overall food craving but did not specifically intensify cravings for any particular type of food or influence food liking. |
| Schulte, 2017 | Cross-sectional;  Participants reported how likely they were to experience a loss of control over their consumption of 30 nutritionally diverse foods and rated each food on five subjective effect report questions assessing substance abuse liability (liking, pleasure, craving, averseness, intensity). | n= 501 participants  37.5% of participants identified  as male (n = 188), 62.1% identified as female (n = 311), and 0.4% identified as other (n = 2). | 34.5 ± 10.8 | 28.8 ± 7.4 | Clustering foods based on FC resulted in two clusters that also appear to separate foods based on processing status. | 14.6%  (n = 73)  Symptom count: 2.39 ± 3.12 | Nutritionally diverse foods were clustered based on loss of control, a feature of addictive-like consumption, and five subjective effect report questions used to assess substance abuse liability. Findings suggest that subjective reports on averseness and taste intensity do not effectively distinguish foods associated with loss of control consumption. |
| Li, 2019 | Case-control;  Fasting blood samples were obtained before and one-month after bariatric surgery. Plasma concentrations of total ghrelin, leptin and insulin were measured.  All clinical measurements were conducted before (baseline) and one month after surgery;  Measurements  - fMRI food-cue-reactivity task with high-calorie and low-calorie food pictures  - MRI acquisition | n= 22  Obesity (n= 19) in the control group  Pre-BS: Patients with obesity who received MRI scan BS;  Post-BS: Patients with obesity who received MRI scan at one month after surgery;  CtrT: Control subjects who received MRI scan at baseline;  CtrRT: Control subjects who  received MRI scan one month after the first scan; | Pre-BS:  26.64 ± 1.83  Post-BS: 26.64 ± 1.83  CtrT: 28.63 ± 2.06  CtrRT: 28.63 ± 2.06 | Pre-BS: 38.11 ± 1.32  Post-BS: 34.03 ± 1.31  CtrT: 35.27 ± 1.01  CtrRT: 35.14 ± 1.04 | Pre-BS:  HC: 65.00 ± 5.26  LC: 46.17 ± 4.56  Post-BS:  HC: 31.48 ± 4.66  LC: 42.74 ± 5.99  CtrT:  HC: 66.67 ± 6.43  LC: 49.17 ± 6.62  CtrRT:  HC: 62.22 ± 4.83  LC: 48.33 ± 4.99 | Symptom count:  Pre-BS: 4.95 ± 0.59  Post-BS: 2.81 ± 0.41  CtrT: 3.37 ± 0.56  CtrRT: 3.42 ± 0.53 | LSG significantly decreased plasma ghrelin levels, FC for high-caloric food, and DLPFC activation in response to high- versus low-caloric food cues in the BS group. Reduced DLPFC activation post-surgery correlated positively with decreased ghrelin levels and FC for high-caloric cues. Additionally, LSG increased connectivity between the right DLPFC and vACC, with greater connectivity linked to larger BMI reductions post-surgery. |
| Schulte, 2019 | Experimental study;  In the taste test task, participants sampled 14 nutritionally diverse foods, each presented in standardized 7g portions in a randomized order. Between samples, participants rinsed their mouths with water. They were instructed to eat slowly and focus on the eating experience. After each portion, participants rated their subjective experience, including craving, liking, pleasure, and satisfaction, using visual analog scales (VAS). | n= 44 women with overweight or obesity | 30.75 ± 4.20  range: 25 - 40 | 33.68 ± 5.46  range: 24.70 - 51.00 | 28.70 on a scale ranging from 0 to 100, although this was significantly  higher (+11.98) for individuals with FA | 38.6% (n=17) | Highly processed foods were strongly linked to subjective experiences suggesting higher addictive potential. Individuals with FA reported lower subjective experience ratings for highly processed foods compared to those without addictive-like eating behaviors. Elevated subjective experience ratings for highly processed foods correlated with increased consumption, especially among individuals with FA. However, no differences in caloric intake between highly processed and minimally processed foods were observed based on YFAS scores. |
| Bach et al., 2021 | Prospective longitudinal study;  They were evaluated using fMRI two weeks before surgery (T0), and at eight (T1) and 24 weeks (T2) post-surgery. During the fMRI sessions, they were exposed to images of food and neutral stimuli. Additionally, participants completed questionnaires assessing their eating behaviors, including the YFAS, BDI.  FC was assessed using a VAS. The 18-minute fMRI task included food stimuli categorized as high-calorie salty (e.g., burger, pizza), high-calorie sweet (e.g., ice cream, cake), and low-calorie options (both salty and sweet, e.g., salad, fruits). | Total:  n= 26 individuals with obesity (65.4% female)  Participants with imaging data:  n= 11 (72.7% female)  From the 26 participants, 21 underwent Roux-en-Y gastric bypass, and 5 underwent vertical sleeve gastrectomy. | Total:  41.46 ± 12.61  Participants with imaging data: 41.18 ± 10.17 | Total:  46.35 ± 6.60  Participants with imaging data: 45.40 ± 4.75 | T0 = 45.19 ± 23.44  T1= 18.55 ± 39.92  T2= 32.45 ± 25.97  Cue-induced craving showed a significant reduction from T0 to T1 (p= 0.027), but no significant increase or decrease from T1 to T2 (p= 0.182). | Total: 2.54 ± 1.88  Participants with imaging data: 3.09 ± 2.02 | Hunger perception and YFAS scores accounted for 50.6% of the variance in % total weight loss at 24 weeks post-surgery in the total sample. In the subgroup with fMRI data (n= 11), food craving induced by visual cues at baseline (pre-surgery) explained 49.6% of the variance in %total weight loss. There was also a significant decrease in amygdala activation and an increase in orbitofrontal cortex activation in response to food images after bariatric surgery. The findings suggest that bariatric surgery may modulate brain areas associated with reward and inhibitory control, contributing to long-term weight loss success. |
| **Food Craving Inventory (FCI)** | | | | | | | |
| Pepino, 2014 | Longitudinal study;  Subjects were studied before and after 20% weight loss induced by BS.  Participants received standardized dietary counseling to achieve approximately 20% total weight loss within six months post-surgery. Post-surgery guidelines included a liquid diet for the first week, followed by a gradual transition over 2–4 weeks to a regular diet of 1000–1200 kcal/day with 1.0 g of protein per kg of body weight daily. Eating behavior assessments conducted pre-surgery were repeated after participants achieved 20% (range 15–28%) weight loss. | (n= 44)  (39 women, 5 men)  FA  group (FA) (n=14)  No FA  group (n= 30) | FA: 42.6 ± 10.9  Non-FA: 43.2 ± 11.1 | 48 ± 8  FA:  Before surgery: 48.2 ± 8.2  After surgery: 38.5 ± 6.9  Non-FA:  Before surgery: 47.5 ± 8.0  After surgery: 37.7 ± 6.6 | FA: 24 ± 18  Non-FA: 16 ± 17 | 32% (n= 14) | No significant differences were observed between FA and non-FA groups in factors such as age, race, education level, or income. Surgery-induced weight loss led to FA remission in 93% of participants who met FA criteria pre-surgery, with no new FA cases emerging post-surgery. The prevalence of FA decreased from 32% to 2%. Additionally, surgery-induced weight loss reduced FC, emotional eating, and external eating behaviors in both groups. |
| Joyner, 2015 | Cross-sectional study;  Participants provided basic demographic information and completed a battery of self-report measures. Self-reported height and weight were used to compute participant BMI (kg/m^2^).  Questionnaires assessed were: YFAS; FCI; EDE-Q. | n= 283  The sample was 15.9% male (n= 45) and 83.0% female  (n = 235), and 3 participants did not report gender. | 34.9 | 28.44 ± 9.00 | The mean total craving score in the sample was 2.09 ± 0.83. Subscale mean scores were: fats (1.75 ± 0.88), fast food fats (2.36 ± 0.99), sweets (2.32 ± 0.99), and carbohydrates (1.91 ± 0.90). | The mean YFAS symptom count score in the current sample was 3.02 ± 2.00 | FC partially mediates the relationship between addictive-like eating and both BMI and binge eating episodes. Cravings for different food types showed distinct mediation effects: cravings for fats mediated the link between addictive-like eating and BMI but not binge eating episodes, while cravings for sweets mediated the relationship between addictive-like eating and binge eating episodes but not BMI. |
| Chao, 2019 | Prospective, single-arm;  The 14-week group behavioral weight loss program included a structured meal replacement diet and consisted of weekly 90-minute in-person lifestyle modification sessions. Groups of 10–15 participants were led by registered dietitians or psychologists. | n= 178 participants  Most of the sample was female (87.6%),  Hypertension (53.9%) and dyslipidemia (46.1%) were the most common comorbidities. | 44.2 ± 11.2 | 40.9 ± 5.9 | FCI total score: 2.3 ± 0.7  Among participants, 83.7% reported cravings for all four food types, 8.4% for three, 1.1% for two, and 2.2% for only one. Notably, only 1.1% reported no cravings for any food types in the past 28 days. | 6.7% (12 of the 178 participants)  The number of FA  symptoms in the total sample was 2.3 ± 1.6 out of a potential seven symptoms. | Participants meeting YFAS criteria for FA reported significantly higher total FC and cravings for sweets, complex carbohydrates/starches, and fast-food fats compared to non-FA participants. Over 14 weeks, participants lost an average of 9.0 ± 0.3% of their initial weight. Higher cravings for sweets and high fats were associated with smaller weekly weight losses. However, individuals with FA lost 6.5 ± 1.2% of their initial weight, a value not significantly different from those without FA. |
| Wiedemann, 2021 | Cross-sectional study;  To evaluate the clinical significance of the proposed three severity specifiers of the YFAS.  Participants completed an online survey to self-report measures assessing FA, eating-disorder psychopathology (Eating Disorder Examination Questionnaire) and FCI. | n= 1854 (67.6% female)  Non-FA: n=1643  Mild: n=40  Moderate: n=55  Severe: n=116 | 36.7 ± 12.2 | 27.6  ± 6.9 | Non-FA: 2.21 ± 0.02  Mild FA: 2.34 ± 0.11  Moderate FA: 2.38 ± 0.09  Severe FA: 2.65 ± 0.07 | YFAS (total sample): 1.56 ±2.50 | Compared to mild, moderate, or severe FA groups, the Non-FA group reported significantly lower levels of shape, weight, and eating concerns, as well as lower global eating-disorder psychopathology. Dietary restraint was also significantly lower in the Non-FA group compared to mild/severe FA groups. The severe FA group showed higher FC scores than the No FA group. Binge-eating episodes significantly increased with greater FA severity across the YFAS groups. |
| Schulte, 2021 | Cross-sectional study;  To examine differences in questionnaires assessing behavioral characteristics of addictive disorders to determine whether a diagnosis of FA reflects unique features within the context of overweight and obesity. | Women (*n*=46) with overweight or obesity  FA: 20  No FA: 26 | 31 ± 4.1 | 33.9 ± 5.5 | Total: 2 ± 0.6  FA: 3 ± 0.6  No FA: 2 ± 0.6 | FA: 7.3 ± 2.0  No FA: 0.2 ± 0.4 | FA appears to represent a distinct phenotype within overweight and obesity. Greater greater emotion dysregulation, impulsivity, and cravings was found in +FA. |
| **Food Craving Questionnaire - Trait and State (FCQ-T/S)** | | | | | | | |
| Davis, 2011 | Case-control;  Adults BMI >30 aged 25–45 were evaluated through face-to-face interviews across three domains commonly used to characterize substance-dependence disorders: clinical comorbidities, psychological risk factors, and abnormal motivation for the addictive substance. | n= 72  FA (n= 18)  Non-FA (n= 54) | FA: 35.3  Non-FA: 33 | FA: 37.5  Non-FA: 38.8 | FA: 177.8 ± 28.2  Non-FA: 127.2 ± 32.3 | 25%  (26,5% of women, and 21,7% of men) | Individuals meeting diagnostic criteria for FA exhibited significantly higher comorbidity with BED, depression, and ADHD compared to age- and weight-matched counterparts. They were more impulsive, emotional, and prone to using food for self-soothing, with higher FC than obese controls. Notably, half of those with BED did not meet FA criteria, and nearly 30% of the FA group were not clinically significant binge eaters. |
| Meule & Kübler, 2012 | Cross-sectional study; | n= 616  The majority of participants  were women (75.8%, n= 467). Almost all participants were students (80.9%) and had German citizenship (95.5%). | 24.5 ± 4.0 | 22.3 ± 3.3 | Results described between groups, without total sample score | FA (n=48)  Non-FA participants (n= 568) | Groups showed no differences in age or BMI. Individuals with FA had higher FCQ-T total scores and elevated scores on all FCQ-T subscales, except for positive reinforcement. Positive reinforcement negatively predicted FA symptoms, while other subscales showed positive associations. An interaction revealed that FA symptoms increased with heightened FC alongside reduced anticipation of positive reinforcement. |
| Meule, 2012a | Cross-sectional study;  Stimuli consisted of 10 pictures per category (savory foods, sweets, and neutral objects). The task included images serving as both targets and distractors. Participants were tested between 10:00 a.m. and 6:00 p.m. (median time: 2:00 p.m.) and were instructed to refrain from eating, smoking, and consuming caffeinated drinks for at least one hour before the experiment. Following informed consent and task instructions, a 15-minute baseline heart rate recording was conducted. | n= 56  Frequent and intense (high cravers, n= 28) and less pronounced  FC (low cravers, n= 28) | 24.12 years ± 3.79)  Low cravers:  24.46 ± 3.69  High cravers:  22.41 ± 3.34 | 22.65 ± 3.19)  Low cravers:  23.79 ± 3.93  High cravers:  22.90 ± 3.07 | FCQ-T  Low cravers:  83.11 ± 19.23  High cravers:  131.43 ± 23.38 | symptom count  Low cravers:  1.11 ± 0.50  High cravers:  2.54 ± 1.35 | High cravers exhibited higher FCQ-T scores and greater eating-related psychopathology, as measured by the YFAS and EDE-Q, compared to low cravers. During a working memory task with high-calorie food cues, participants showed slower reaction times and more omission errors, especially with savory food images. Post-task, all participants reported increased FC, with the effect being significantly more pronounced in those who frequently and intensely experience FC. |
| Meule, 2012b | Cross-sectional;  An online screening was conducted to recruit high and low-food cravers. The screening homepage included the subscale lack of control over eating of the FC Trait (FCQ-T; see below). This subscale represented a significant feature of FC and was chosen to keep the screening succinct.  After providing instructions and signing informed consent, a 10 min baseline heart rate recording was conducted. Then, participants performed a working memory task with pictures of food and neutral stimuli, which is reported elsewhere. The group HRV-biofeedback received the Intervention for four weeks. After four weeks, the very same routine was conducted for the first measurement. | n=56, classified in:  High cravers  (n= 28)  Low cravers (n= 28)  They were divided in three groups:  **CB:** HRV-biofeedback (half of the high cravers: n= 14)  **CC:** No intervention (another half of the high cravers: n= 14)  **NCC:** No intervention (non-craving control group: n= 28) | 24.12 ± 3.79 | 22.65 ± 3.19 | Pre-Measurement:  CB: 130.57 ± 18  CC: 132.29 ± 28.40  NCC: 83.11 ± 19.23  Post-Measurement:  CB: 113.57 ± 15.30  CC: 132.00 ± 34.89  NCC: 77.00 ± 18.52 | Pre-Measurement:  CB: 2.14 ± 1.03  CC: 2.93 ± 1.54  NCC: 1.11 ± 0.50  Post-Measurement:  CB: 1.79 ± 1.19  CC: 2.93 ± 1.73  NCC: 1.14 ± 0.53 | High food cravers showed reductions in subjective FC and eating- and weight-related concerns following HRV-biofeedback training. Notably, FC associated with lack of control, food preoccupation, and guilt significantly decreased in the biofeedback group. While some FC aspects did not show statistically significant changes, effect size analyses revealed medium-to-large reductions across all FC subscales. Additionally, a decrease in FCQ-T total scores indicated an overall reduction in FC. |
| Davis et al., 2013 | Cross-sectional study;  Eating-behavior questionnaires were completed and a blood sample was taken for genotyping.  Multilocus genetic profile (MLGP) scores were based on six known  dopamine-related polymorphisms (DRD2 Taq1A, DRD2 −141C Ins/Del, DAT1 VNTR, COMT Val158Met, C957T, rs12364283). | Total: n= 120   F: (n= 21  Non-FA: n= 99 | FA: 34.7 ± 5.9  Non-FA:32.5 ± 6.6 | FA: 35.5 ± 7.3  Non-FA: 33.1 ± 8.9 | FA: 176.8 ± 27.6  Non-FA: 115.9 ± 34.4. | 17.5% | The MLGP score was higher in individuals with YFAS-diagnosed FA and positively correlated with binge eating, FC, and emotional overeating. Mediation analysis revealed that enhanced dopamine signaling, inferred from the MLGP score, was significantly stronger in the FA group compared to controls, with FC, binge eating, and emotional overeating mediating this relationship. |
| Davis & Loxton, 2014 | Experimental study;  A venous blood sample was taken at the hospital laboratory, and the questionnaire package was completed at home and returned at a later date. | Total: n= 145  FA: n= 25  Non-FA: n= 114  They were divided in three groups, according to their alleles (GG, GA, AA); | GG: 31.9 ± 6.5  GA: 33.2 ± 6.2  AA: 32.6 ± 6.6 | GG: 31.1 ± 8.0  GA: 32.2 ± 8.6  AA: 33.9 ± 8.4 | not presented in detail | YFAS Symptom Score  GG: 3.1 ± 2.1  GA: 2.2 ± 1.7  AA: 2.9 ± 2.0 | This study preliminarily demonstrated a relationship between brain opioid signaling strength and individual differences in hedonic responsiveness to tasty, high-caloric foods. The three hedonic-responsiveness variables—FC, hedonic eating, and high fat/sugar preference—were moderately to highly intercorrelated, as anticipated. |
| Meule, 2014a | Cross-sectional study;  If task performance was associated with self-reported impulsivity, current food deprivation, BMI, and self-report measures related to overeating. | n= 94  FA (n= 38)  Non-FA (n= 56) | 39.94 ± 11.59  FA: 39.29 ± 9.77  Non-FA  group: 40.38 ± 12.74 | 50.73 ± 9.02  FA: 50.89 ± 8.08  Non-FA: 50.62 ± 9.68 | 111.58 ± 38.78  FA  group: 135.73 ± 31.90  Non-FA: 93.85 ± 33.63  - FCQ-S: 30.88 ± 12.25  FA group: 31.94 ± 12.21  Non-FA: 30.10 ± 12.35 | 40%  symptom count: 3.39 ± 1.75 | FA was associated with trait FC, higher eating disorder psychopathology—including eating, weight, and shape concerns—and more frequent binge episodes. FA scores also correlated with higher depression scores. While the FA group showed marginally lower AUDIT scores than the no-FA group, these scores were not correlated with FA symptoms. Symptomatology of FA was specifically linked to attentional impulsivity, but not to motor or non-planning impulsivity. In contrast, AUDIT scores were correlated with motor and non-planning impulsivity, but not with attentional impulsivity. Notably, higher impulsivity was associated with increased alcohol use only in non-food-addicted individuals, but not in those diagnosed with FA. |
| Meule, 2014b | Cross-sectional study;  Participants were tested between 9:00 a.m. and 5:30 p.m. All participants were asked not to consume food, caffeine, nicotine, or alcohol at least 3h before the experiment. After participants had performed the stop-signal task SST, they immediately filled out the FCQ-S and reported the hours that had elapsed since their last meal. | n= 50 | 22.32 ± 3.03 | 21.45 ± 2.67 | 44.78 ± 10.56 | symptom count  1.56 ± 1.05 | Participants exhibited slower reaction times in food trials compared to neutral trials. Increased reaction times in response to food cues were associated with heightened FC after the task and elevated self-reported hunger, indicating an effect of food cues beyond simple categorization. Reduced inhibitory control in response to food cues correlated with increased FC post-task, specifically an intensified desire to eat and a perceived lack of control over eating. While motor impulsivity was linked to poorer inhibitory performance during the task, it showed no direct association with current FC. |
| Meule, 2015 | Cross-sectional study;  Participants: Adolescent inpatients recruited within the first 2 weeks of weight-loss treatment in a rehabilitation hospital in Germany. | n= 50  n= 31 (62%) girls  n= 19 (38%) boys | 16.50 ± 1.84, range: 14–21) | 36.80 ± 6.18, range: 26.17– 56.32 | 107.98 ± 37.85 | 38%  symptom count:  3.38 ± 2.11, range: 0–7 | The most common FA symptoms were a persistent desire or repeated unsuccessful attempts to reduce consumption, tolerance, and continued overeating despite physical or psychological issues. Individuals with a YFAS diagnosis exhibited higher eating, weight, and shape concerns, reported more binge eating episodes and FC experiences, had elevated depression symptoms, and scored higher on attentional and motor impulsivity compared to those without a YFAS diagnosis. These variables were also positively correlated with the YFAS symptom count, with the exception that symptom count additionally correlated with non-planning impulsivity. |
| Niemiec, 2016 | Cross-sectional;  The proposed 14-item Obsessive Compulsive Eating Scale (OCES) is based on the existing and well-validated Obsessive Compulsive Drinking Scale, a self-report measure of obsessive thoughts about drinking and compulsive behaviors directed towards alcohol consumption. The OCES was administered to 224 respondents.  Only participants that reported not having any current avoidance of a specific food or food group.  were recruited to this study. | Total participants: n= 224  Women: 57.4%  Men: 42.6%  Groups:  - Total (n= 224)  - Women (n= 128)  - Men (n= 95) | Total:  18.89 ± 1.91  Data was not shown for men and women | Total participants:  25.17 ± 4.19  Women:  24.97 ± 4.12  Men:  25.81 ± 4.22 | **FCI**  Total participants: 2.27 (0.65)  Women: 2.31 (0.60)  Men: 2.22 (0.71)  **FCQ-T-r**  Total participants:  37.61 ± 12.4  Women: 41.00 ± 12.24  Men: 33.12 ± 11.19 | Total participants: 20.74 ± 11.64  Women: 22.10 ± 11.84  Men: 18.95 ± 11.19 | The OCES is a brief and flexible self-report tool designed to assess the cognitive foundations of FC. This study demonstrated its excellent psychometric properties, highlighting its potential as a valuable predictor of binge and overeating episodes. Subscale scores effectively distinguished between binge eaters and non-binge eaters, as well as between individuals categorized as overweight and not overweight. Additionally, OCES scores were positively associated with measures of general and specific FC, eating disorder pathology, FA, restrained eating, weight dissatisfaction, and impulsivity. |
| Giel, 2017 | Randomized controlled proof-of-concept study;  Patients with binge eating disorder (BED) were randomly assigned to three sessions of food-specific inhibition training (FIT) or a control condition (CC). In both conditions, high-caloric food images were displayed in peripheral vision on a computer screen while gaze behavior was monitored. The FIT group was instructed to suppress the urge to look at these images (perform antisaccades), while the CC group was free to explore them. Self-reported FC, FA, and wanting/liking of food images were measured before and after the intervention, with a follow-up assessment conducted four weeks later. | n= 20 patients with BED, separated in two groups:  CC (control condition)  FIT (food-specific inhibition training)  Only female patients took part in the study | 36.6 ± 11.9. | 29.6 ± 6.3 | At baseline:  FIT: 43.5 ± 11.4  CC: 37.7 ± 13.4  Follow-up:  FIT: 44.6 ± 14.2  CC: 39.8 ± 15.6 | At baseline:  FIT: 3.4 ± 1.8  CC: 3.4 ± 1.4  Follow-up: 3.4 ± 1.3  CC: 3.5 ± 1.8 | Patients of the training group significantly improved inhibitory control towards high-caloric food stimuli. Both groups reported a significantly lower number of binge eating episodes in the last four weeks after the termination of the study. No changes were found in FC, FC, liking, and wanting ratings. |
| Meule, 2017 | **Study 1**  Participants were recruited in February and March 2015 via students' mailing lists at various universities.  **Study 2**  Data from bariatric surgery candidates were obtained between January and October 2015 at Hannover Medical School. Participants were recruited within the routine preoperative psychiatric evaluation. | **Study 1**  Total participants:  n= 455; 89% were female (n = 405)  Groups:  FA (n= 44)  Non-FA (n= 411)  **Study 2**  Total participants: n= 138; 78.3% were female (n= 108)  Groups:  FA (n= 63)  Non-FA (n= 70) | **Study 1**  Total participants:  25.57 ± 6.97  FA:  27.27 ± 8.49  Non-FA  25.38 ± 6.77  **Study 2**  Total participants:  39.52 ± 10.71  FA:  39.83 ± 10.60  Non-FA  39.61 ± 10.92 | **Study 1**  Total participants:  22.32 ± 3.65  FA:  23.89 ± 5.29  Non-FA  22.15 ± 3.40  **Study 2**  Total participants:  48.80 ± 7.08  FA:  49.46 ± 7.51  Non-FA:  48.14 ± 6.79 | **Study 1**  FA: 61.00 ± 14.57  Non-FA: 31.52 ± 11.06  **Study 2**  FC was not measured | **Study 1**  9.67%  **Study 2**  45.65% | **Study 1**  Participants with a YFAS 2.0 diagnosis exhibited higher BMI, FCQ-T-r, and attentional impulsivity scores, more binge eating days, and lower PSRS scores compared to those without a diagnosis. The number of YFAS 2.0 symptoms positively correlated with BMI, FCQ-T-r, and attentional impulsivity, while binge eating days showed a negative correlation with PSRS scores. Additionally, age, motor impulsivity, and total BIS-15 scores were positively associated with the number of YFAS 2.0 symptoms.  **Study 2**  Participants with a YFAS 2.0 diagnosis reported more binge eating days and higher eating, weight, and shape concerns compared to those without a diagnosis, despite similar BMI and restraint scores between groups. YFAS 2.0 symptoms positively correlated with binge eating days, eating, weight, and shape concerns, but not with BMI or restraint scores. Additionally, attentional impulsivity was positively associated with the number of YFAS 2.0 symptoms. |
| Meule, 2018 | Cross-sectional study;  Receiver-Operating-Characteristic analysis was used to determine the sensitivity and specificity of scores on the FC Questionnaire-Trait-reduced (FCQ-T-r) for discriminating between individuals with (n = 43) and without (n=389) FA as assessed with the YFAS. | n=432 participants  88.4% female (n = 382). | 25.6 ± 7.09 | 22.3 ± 3.70, range: 12.2–42.5 | 34.5 ± 14.5 | 10% (n = 43)  Mean number of endorsed FA  symptoms was 1.32 ± 2.52, range: 0–11 and 43 participants  (10.0%) were classified as FA. | In the ROC analysis, the area under the curve was 0.925 (SE = 0.03, p < 0.001, 95% CI: 0.872–0.979), indicating excellent discrimination between individuals with and without FA. Sensitivity and specificity values exceeded 80% at FCQ-T-r scores between 41 and 52, with sensitivity at 84.9% and specificity at 92.5% at a score of 50. Findings suggest a potential cut-off range between 32 and 54 for differentiating FA presence and absence. A cut-off score of 50 demonstrated high sensitivity and specificity and corresponds to one standard deviation (SD = 15) above the mean FCQ-T-r score (M = 35) in this study. |
| Leong, 2018a | Randomized, double-blind, placebo-controlled;  Participants underwent six sessions of either Infraslow Neurofeedback (ISF-NF) or placebo over three weeks. Resting-state brain activity was measured using EEG. The first session lasted 10 minutes, while the remaining five sessions were 20 minutes each. Two days after the final session, participants completed the same set of questionnaires and underwent another resting-state EEG assessment. | n= 21  ISF-NF (n= 11)  Placebo (n= 10)  All participants were women | ISF-NF: 44.0 ± 13.2  Placebo: 42.0 ± 14.7 | ISF-NF: 33.6 ± 8.5  Placebo: 33.4 ± 6.2 | ISF-NF vs. placebo  Intense desire to eat: 3.8 ± 1.0; placebo: 3.8 ± 0.6  Anticipation of relief from negative states: 3.6 (0.7); placebo: 3.4 (0.9)  Craving as a physiological state: 3.2 (0.9); placebo: 3.2 (0.7)  Anticipation of positive reinforcement: 3.5 (0.8); placebo: 3.5 ± 0.8  Lack of control over eating: 3.6 (1.2); placebo: 3.6 ± 0.8 | ISF-NF: 4.9 ± 1.6  Placebo: 5.1 ± 1.4 | Findings suggest that source localized ISF-NF results in electrophysiological changes and may be associated with reduced FC.  There were no significant correlations between changes in FC measures and infraslow  brain activity in the PCC. |
| Leong, 2018b | Randomized, double-blind, parallel trial);  Participants were randomized into real or sham HD-tPNS groups. At baseline (T0), FC was assessed using the FCQ-S, and resting-state brain activity was measured via EEG after overnight fasting. Both groups received HD-tPNS or sham treatment three times weekly for two weeks, totaling six sessions. Assessments were repeated after two days (T1), at week four (T2), and at week six (T3) post-treatment, all following overnight fasting. | n= 16  Groups:  Sham HD-tPNS (n= 8)  Real HD-tPNS (n= 8) | Sham HD-tPNS:41.1 ± 12.7  Real HD-tPNS: 38.4 ± 11.3 | Baseline:  Sham HD-tPNS: 40.4 ± 11.2  Real HD-tPNS: 39.8 ± 4.9  T3  Sham HD-tPNS:  Real HD-tPNS: | Was measured by FCQ-S:  Baseline:  Sham HD-tPNS: 3.24 ± 0.87  Real HD-tPNS: 3.63 ± 0.37  T3  Sham HD-tPNS: 3.08 ± 0.71  Real HD-tPNS: 2.84 ± 0.86 | Sham HD-tPNS: 4.8 ± 1.4  Real HD-tPNS: 5.4 ± 1.6 | Findings showed a significant decrease in both groups over time for total score and 'lack of control over eating'(placebo effect could result from the expectation of a therapeutic benefit). Preliminary findings showed that HD-tPNS stimulation of the Anterior cingulate cortex can suppress FC in individuals with obesity who show signs of FA. |
| Wang, 2019 | Cross sectional;  A web-based survey was conducted in 2017. The 208 participants were recruited from  multiple sources, including the psychology department’s online participant pool, informal requests, and advertising on social media. | Total participants: n= 208)  Women: n= 167 (80.3%)  Men: n= 41 (19.7%)  Groups:  FA (n= 30)  Non-FA (n= 178) | Total s: 26.82 ± 7.89  FA: 26.87 ± 8.62  Non-FA:  26.81 ± 7.78 | Total participants: 27.53 ± 12.21  FA:  31.62 ± 13.46  Non-FA:  26.85 ± 11.90 | Was measured by FCQ-T-r  Total participants:  41.32 ± 16.66  FA:  65.17 ± 15.93  Non-FA:  37.30 ± 13.06 | FA: 14.4%  Non-FA  85.6% | This study aimed to examine the prevalence of FA in a community sample and identify differences in demographics, BMI, eating-related behaviors, body perception, and personality traits, as well as assess symptom severity and its association with physical and psychological variables. FA prevalence was found to be 14.4%, supporting the validity of the FA construct. Participants with three or more FA symptoms were more likely to be female and have a higher BMI compared to those with fewer symptoms. |
| El Archi, 2020 | Cross-sectional study;  A total of 372 non-clinical participants completed the online questionnaire: 76 women were considered to be at risk for an eating. | n= 76 at risk for an eating disorder.  Anorexia nervosa (AN) (n= 28)  Bulimia nervosa (BN) (n= 19)  Binge eating Disorder (BED) (n= 29): | 26.8 ± 9.8  AN: 27.07 ± 11.04  BN: 26.42 ± 10.20  BED: 26.72 ± 8.57 | 23.0 ± 8.0  AN: 16.94 ± 1.31  BN: 24.60 ± 6.89  BED: 27.89 ± 8.71 | Mean FCQ-T-r score was 61.2 ± 17.4 | AN: 35.7%  BN: 73.7%  BED: 62.1% | Results showed lower FC intensity in individuals at risk for AN compared to those at risk for BN or BED. Only the BED group demonstrated a positive correlation between FC and BMI. These findings suggest that while individuals with AN exhibit excessive cognitive control, it does not eliminate FC but may reduce its physiological and cognitive manifestations, explaining the lower FC intensity in this group. In contrast, FC was linked to higher BMI in individuals at risk for BED. |
| Oliveira, 2020 | Cross-sectional  Patients seeking Treatment for FA with moderate-to-severe FA (assessed by the YFAS 2.0) completed an Interview (MINI), Binge Eating Scale (BES), Food Cravings Questionnaire—Trait version (FCQ-T), Beck Depression Inventory (BDI-II) e Beck Anxiety Inventory (BAI), WHOQOL-brief. | n=46 (80.4% women) | 43.28 ±10.82 | - | 166,39 ± 30,27 | 9.12 ± 1.81 | FA was positively associated with binge eating severity, anxiety symptoms, and psychological, physical, and social impairment but showed no significant correlation with FC, depression, or the environment domain of the WHOQOL brief. Regression analyses, controlling for binge eating severity, FC, depression, and anxiety, identified FA as a significant predictor of social impairment. These findings suggest that individuals seeking treatment for FA often present with notable comorbidities, particularly anxiety disorders. |
| Haghighinejad et al., 2021 | Cross-sectional;  To investigate the validity and reliability of the Persian version of the YFAS 2.0, determine the prevalence of FA in an Iranian population, and identify potential correlations between FA, FC, BMI, and age, using the YFAS 2.0 and the FCQ-T-r. | Total: n= 330 (65.2% female)  FA: n= 22 (77.3% female)  Non FA:  n= 308 (64.3% female) | Total: 33.3 ± 10.3 (range: 18–73)  FA:31±10.5  Non-FA: 33.4±10.3 | Total: 24.2 ± 4.4  FA: 27.4 ± 7.4  Non-FA: 24 ± 4 | Total: 30.2 ± 13.6  FA: 50.1 ± 17.6  Non-FA: 28.8 ± 12.1 | Prevalence of FA was 6.7% (n=22): 2.7% severe; 1.2% moderate; 2.7% mild.  Total: 0.9±1.8  FA: 4.9±2.8  Non-FA: 0.6±1.3 | The Persian version of the YFAS 2.0, according to this study, is a valid and reliable measure for assessing FA in the Iranian population. Results indicated that BMI and FCQ-T-r scores showed a positive correlation with the number of FA symptoms. Age showed a negative correlation with the number of symptoms. ROC curve analysis suggested a cutoff point of 32.5 on the FCQ-T-r to identify FA, with a sensitivity of 91% and specificity of 72%. |
| Sommer et al., 2021 | Cross-sectional;  To assess the differences and similarities between patients with OB with BED and OB individuals without BED, participants completed a series of questionnaires assessing eating-related symptoms, including EDE-Q, SEES, SSE, DEBQ, PSRS, FCQ-T, YFAS 2.0. They also assessed general psychopathology, as well as childhood experiences. | n=131 (68,70% female)  OB-BED patients: n=37  OB controls: n=50  Normal-weight controls: n=44 | 42.7 (range: 21 - 82) | OB-BED: 46.3 ± 9.3  OB: 42.9 ± 9.1  Controls: 22.5 ± 1.6 | FC total scores differed significantly between groups,  F(2, 121) = 58.02, p< 0.001, h2 = 0.49, with OB-BED scoring higher than OB and CO,  ps < 0.001, and OB scoring higher than CO, p< 0.001. | FA symptoms differed significantly across groups, F(2, 128)= 45.66, p< 0.001, n^2^= 0.42, with OB-BED scoring higher than OB and CO, ps< 0.001, and OB scoring higher than CO, p< 0.001. | Patients with OB and BED showed significantly higher scores across all assessments, including depression, emotional and stress eating, FC and FA, compared to the other two groups. However, no significant differences were found between patients with obesity and BED and OB individuals without BED regarding childhood trauma or attachment styles. The results indicate that OB with and without BED share common characteristics, such as adverse childhood experiences and certain aspects of general psychopathology, but also exhibit important differences. |
| Bruzas, 2022 | Cross-sectional  34 individuals diagnosed with BED enrolled in a randomized double-blind placebo-controlled trial of liraglutide 3.0 mg/d for the treatment of BED completed the Loss of Control Over Eating Scale (LOCES), the Eating Disorder Examination, and measures of eating behavior, mood and quality of life | n=34 (65% women) | 41.2 ± 10.1 | 36.2 ± 6.5 | FCQ-T 160.89 ± 34.55  FCQ-S 50.55 ±10.95 | 6.50 (3.19) | Greater loss of control (LOC) showed a stronger independent association with eating psychopathology, shape dissatisfaction, hunger, FC, and FA symptoms, while larger binge size was more strongly linked to weight concern and lower general and social quality of life. Both LOC and binge size were associated with eating concerns but not with depression or BMI. These findings highlight LOC and binge size as key psychosocial treatment targets for individuals with BED. |
| [Ghanbari](https://jeatdisord.biomedcentral.com/articles/10.1186/s40337-022-00689-5#auth-Nikzad-Ghanbari), 2022 | Cross-sectional  451 iranian students completed online the YFAS 2.0, Depression, Anxiety, and Stress Scale-21 (DASS-21), and Food Craving Questionnaire-Trait, reduced (FCQ-T-r), in order to investigate the psychometric assessment of  the Persian translation YFAS 2.0 and validate this version | n=451 (65.0% women) | 25.8 ±9.3 | - | 29.98 ± 15.18) | 64.63 ± 30.65 | The YFAS’s 2.0 presented positive correlations with three DASS-21 subscales (depression, stress and anxiety) and with FC Questionnaire-Trait reduced (FCQ-T-r) (desire, hunger and reinforcement), which indicates acceptable convergent validity of the Persian translation version of YFAS’s 2.0 |
| **General Food Cravings Questionnaire (GFCQ)** | | | | | | | |
| Davis, 2014 | Randomized, double-blind, cross-over design;  The 2.5-hour session included baseline mood assessments, followed by repeated evaluations every 15 minutes after ingesting a methylphenidate capsule. After 1 hour and 15 minutes, participants held their favorite snack, answered appetite rating questions, and completed a craving questionnaire. They were then allowed to eat as many snacks as they desired. | 136 participants (women= 92;  male= 44)  Women: 67,65%  Male: 32.35%  Diagnostic according to YFAS:  FA: 23  Non-FA: 113 | FA: 33.9 ± 5.9  Non-FA 32.4 ± 6.6 | FA: 34.6 ± 7.0  Non-FA: 33.8 ± 8.4 | Graphic representation | 16.9% | Results demonstrated significant eating-related differences in response to a snack-food challenge between those diagnosed with FA and the non-diagnosed control group. The former reported stronger FC and greater appetite ratings following  a taste of their favorite snack and these differences remained stable in the placebo and the methylphenidate conditions.  To summarize, in response to the methylphenidate challenge, the FA group appeared resistant to the typical appetite-suppression effects of this drug. |
| Ravichandran et al., 2021 | Cross-sectional study;  To identify sex-related differences in brain network connectivity in individuals who meet the diagnostic criteria for FA. Participants were divided into groups with and without food addiction. FA and FC were assessed using the YFAS and GFCQT-r questionnaires, as well as resting-state functional magnetic resonance imaging. Functional connectivity between different brain networks was then analyzed. | n = 150 (68,67% female)  FA: 40  No FA: 110  FA was based on YFAS score ≥ 3 together with clinically impairment or distress | FA:  30.35 ± 9.82  Non-  FA: 33.34 ± 10.69 | All participants had a BMI ≥ 25 | GFCQT-r Total:  FA: 50.10 ± 14.27  Non-FA: 28.51 ± 12.61 | YFAS symptom count:  FA: 4.10 ± 1.39  Non-FA: 1.06 ± 0.58 | Individuals with FA exhibited heightened brain connectivity between the brainstem and reward regions, including the mesencephalic reticular formation, orbital frontal gyrus, and bilateral orbital gyri, with stronger salience network connectivity observed in women. This altered connectivity was positively associated with food cravings (FC). These findings suggest that disruptions in reward-related brain networks may contribute to addictive eating behaviors and uncontrolled overeating. |
| **Neurophysiological response** | | | | | | | |
| Mallorquí-Bagué, 2020 | Cross-sectional study;  Emotional regulation, dysregulation, FA and ED symptoms were accessed through questionnaires. Also, Mini International Neuropsychiatric Interview (MINI) was administered.  Participants completed a computerized task during EEG recording, where they were instructed to down-regulate negative emotions or FC. | AN (20)    Control (20) | AN: 22.7 ± 6.51 (range 18 - 43)  Control (health): 21.0 ± 5.12 (range: 18 - 39) | AN: 16.63 ± 1.06    Control: 20.72 ± 1.78 | Neurophysiological response | AN-R (66.7%)    AN-BP (75%) | LPP amplitudes were significantly reduced during FC down-regulation in both groups. Regardless of task conditions, individuals with AN exhibited smaller P300 amplitudes compared to controls. In the control group, self-reported use of reappraisal strategies positively correlated with LPP amplitudes during emotional regulation tasks, while suppressive strategies showed a negative correlation. |

**Note:** **FCI**: Food Craving Inventory; **EDE**: Eating Disorder Examination; **EDE-Q**; Eating Disorder Examination Questionnaire; **DEBQ**: Dutch Eating Behavior Questionnaire; **SSES:** Salzburg Stress Eating Scale; **SEES:** Salzburg Emotional Eating Scale; **BDI-II:** Beck Depression Inventory, 2nd Edition; **BIS-15:** Barratt Impulsiveness Scale – short form; **PSRS:** Perceived Self-Regulatory Success in dieting; **OCES:** Obsessive Compulsive Eating Scale; **EAT-26:** Eating Attitudes Test; **BIS-11:** Barratt Impulsiveness Scale; **fMRI:** Functional magnetic resonance imaging.
