# Supplementary material for: Questioning the validity of food addiction: a critical review
Source: Front Behav Neurosci. 2025 Jul 30;19:1562185. doi: 10.3389/fnbeh.2025.1562185 (PMC12343691; doi:10.3389/fnbeh.2025.1562185)
Supplement: Supplementary file 2 [file Table_2.docx]

**Supplementary Table 2.**  Summary of instruments utilized to assess FC and FA in the included studies.

| **Construct assessed** | **Instrument** | **Purpose/Description** | **References** |  |
| --- | --- | --- | --- | --- |
| **FC** | | | |  |
| Sensory intensity and perception of FCs | General Labeled Magnitude Scales (gLMS) | Although the gLMS is not a direct measure of FC, it may be relevant to it due to its focus on assessing the intensity of sensory perceptions, such as taste, smell, and texture. The scale captures how individuals perceive these stimuli, which can be influenced by or influence states of craving. | Polk, 2017 |  |
| Intensity of FC | Visual Analogue Scale (VAS) | Consists of a straight line (typically 10 cm) with two endpoints representing extremes of a sensation (e.g., "No craving" to "Strongest craving imaginable"). Used to assess the intensity of a sensation or emotional state. | Gearhardt, 2014 ; Schulte & Gearhardt, 2017; Li et al., 2019; Schulte et al., 2019; Bach et al., 2021 |  |
| FC (frequency and intensity) | Food Craving Inventory (FCI) | A validated and reliable instrument, evaluates cravings over the past 28 days, offering both total and subscale scores across categories such as high fat, sweets, complex carbohydrates/starches, and fast-food fats (White et al., 2002). This dual focus on state and trait dimensions makes the FCI a versatile tool for understanding both immediate and habitual craving patterns. | Pepino et al., 2014; Joyner et al., 2015; Chao et al., 2019; Wiedemann et al., 2021; Schulte & Gearhardt, 2021. |  |
| FC (trait and state) | Food Craving Questionnaire — Trait (FCQ-T)/ reduced  Food Craving (State)/ reduced | Are widely used tools for assessing the multidimensional nature of FC. Introduced by Cepeda-Benito et al. (2000), they provide insights into both state (current cravings) and trait (long-term patterns) aspects of craving.  FCQ-T: Evaluates stable, long-term patterns as a behavioral trait.  FCQ-S: Assesses FC in specific contexts or moments, capturing real-time states. | Davis et al., 2011; Meule & Kübler, 2012; Meule, 2012a; Meule, 2012b; Davis et al., 2013; Davis & Loxton, 2014; Meule, 2014b; Meule et al., 2015; Niemiec et al., 2016; Giel et al., 2017; Meule, 2018; Leong et al., 2018a; Leong et al., 2018b; Wang & Lopez-Fernandez, 2019; El Archi et al., 2020; Oliveira et al., 2020; Haghighinejad et al., 2021; Sommer et al., 2021; Bruzas et al., 2022; [Ghanbari](https://jeatdisord.biomedcentral.com/articles/10.1186/s40337-022-00689-5#auth-Nikzad-Ghanbari) et al., 2022 |  |
| FC (frequency and context) | General Food Cravings | Examines broad characteristics, including frequency and context. | Davis & Loxton, 2014; Ravichandran et al., 2021; Mallorquí- Bagué et al., 2020. |  |
| **FA** | | | |  |
| FA | Yale Food Addiction Scale (YFAS)/ Modified Yale Food Addiction Scale (mYFAS) | Assesses FA based on DSM-IV criteria for substance use disorders. Initial version published in 2009 by Gearhardt, Corbin, and Brownell (2009), and later updated according to DSM-5 criteria (Gearhardt, Corbin, & Brownell, 2016). Both versions of the scale, along with shortened versions such as the modified Yale Food Addiction Scale (mYFAS), have been widely employed in research settings. | Polk, 2017; Gearhardt, 2014; Li et al., 2019; Bach et al., 2021; Pepino et al., 2014; Joyner et al., 2015; Chao et al., 2019; Davis et al., 2011; Meule & Kübler, 2012; Meule, 2012a; Meule, 2012b; Davis et al., 2013; Davis & Loxton, 2014; Meule, 2014a; Meule, 2014b; Meule et al., 2015; Niemiec et al., 2016; Giel et al., 2017; Leong et al., 2018a; Leong et al., 2018b; Wang & Lopez-Fernandez, 2019; Ravichandran et al., 2021. |  |
|  |  |  |  |  |
|  |  |  |  |  |
|  | YFAS 2.0/mYFAS 2.0 | Updated version of YFAS, incorporating DSM-5 criteria, with a focus on compulsive eating behaviors. | Schulte & Gearhardt, 2017; Schulte et al., 2019; Wiedemann et al., 2021; Schulte & Gearhardt, 2021; Meule, 2017; Meule, 2018; El Archi et al., 2020; Oliveira et al., 2020; Haghighinejad et al., 2021; Sommer et al., 2021; Bruzas et al., 2022; [Ghanbari](https://jeatdisord.biomedcentral.com/articles/10.1186/s40337-022-00689-5#auth-Nikzad-Ghanbari) et al., 2022; Mallorquí-Bagué et al., 2020. |  |
|  |  |  |  |  |
|  |  |  |  |  |

**References cited in the table:**

Bach, P., Grosshans, M., Koopmann, A., Pfeifer, A. M., Vollstädt-Klein, S., Otto, M., et al. (2021). Predictors of weight loss in participants with obesity following bariatric surgery - a prospective longitudinal fMRI study. *Appetite* 163:105237. doi: 10.1016/j.appet.2021.105237

Bruzas, M. B., Tronieri, J. S., Chao, A. M., Jones, E., McAllister, C., Gruber, K., et al. (2022). Binge size and loss of control as correlates of eating behavior and psychopathology among individuals with binge eating disorder and higher weight. *J. Behav. Med*. 45, 603–612. doi: 10.1007/s10865-022-00312-7

Cepeda-Benito, A., Gleaves, D. H., Williams, T. L., and Erath, S. A. (2000). The development and validation of the State and Trait Food‑Cravings Questionnaires. *Behav. Ther*., 31, 151–173. doi: 10.1016/S0005-7894(00)80009-X

Chao, A. M., Wadden, T. A., Tronieri, J. S., Pearl, R. L., Alamuddin, N., Bakizada, Z. M., et al. (2019). Effects of addictive-like eating behaviors on weight loss with behavioral obesity treatment*. J. Behav. Med*. 42, 246–255. doi: 10.1007/s10865-018-9958-z Davis & Loxton, 2014;

Davis, C., Curtis, C., Levitan, R. D., Carter, J. C., Kaplan, A. S., and Kennedy, J. L. (2011). Evidence that “food addiction” is a valid phenotype of obesity. *Appetite* 57, 711–717. doi: 10.1016/j.appet.2011.08.017

Davis, C., Loxton, N. J., Levitan, R. D., Kaplan, A. S., Carter, J. C., and Kennedy, J. L. (2013). “Food addiction” and its association with a dopaminergic multilocus genetic profile. *Physiol. Behav*. 118, 63–69. doi: 10.1016/j.physbeh.2013.05.014

El Archi, S., Brunault, P., Ballon, N., Réveillère, C., and Barrault, S. (2020). Differential association between food craving, food addiction and eating-related characteristics in persons at risk for eating disorders. *Eur. Rev. Appl. Psychol*. 70:100513. doi: 10.1016/j.erap.2019.100513

Gearhardt, A. N., Corbin, W. R., and Brownell, K. D. (2009). Preliminary validation of the Yale food addiction scale. *Appetite* 52, 430–436. doi: 10.1016/j.appet.2008.12.003

Gearhardt, A. N., Corbin, W. R., and Brownell, K. D. (2016). Development of the Yale Food Addiction Scale Version 2.0. *Psychol. Addict. Behav*., 30, 113–121. doi: 10.1037/adb0000136

Gearhardt, A. N., Rizk, M. T., and Treat, T. A. (2014). The association of food characteristics and individual differences with ratings of craving and liking. *Appetite* 79, 166–173. doi: 10.1016/j.appet.2014.04.013

Ghanbari, N., Nooripour, R., Firoozabadi, A., Var, T. S. P., Wisniewski, P., and Hosseini, S. R. (2022). Psychometric assessment of Persian translation of Yale food addiction scale version 2.0 (YFAS 2.0) in Iranian college students*. J. Eat. Disord*. 10:158. doi: 10.1186/s40337-022-00689-5

Giel, K. E., Speer, E., Schag, K., Leehr, E. J., and Zipfel, S. (2017). Effects of a food-specific inhibition training in individuals with binge eating disorder-findings from a randomized controlled proof-of-concept study*. Eat. Weight Disord*. EWD 22, 345–351. doi: 10.1007/s40519-017-0371-3

Haghighinejad, H., Tarakemehzadeh, M., Jafari, P., Jafari, M., Ramzi, M., and Hedayati, A. (2021). Persian version of the Yale food addiction scale 2.0: psychometric analysis and setting cutoff point for the food cravings questionnaire-trait-reduced. *Psychiatry Investig*. 18, 179–186. doi: 10.30773/pi.2020.0198

Joyner, M. A., Gearhardt, A. N., and White, M. A. (2015). Food craving as a mediator between addictive-like eating and problematic eating outcomes. *Eat. Behav*. 19, 98–101. doi: 10.1016/j.eatbeh.2015.07.005

Leong, S. L., De Ridder, D., Vanneste, S., Sutherland, W., Ross, S., and Manning, P. (2018b). High definition transcranial pink noise stimulation of anterior cingulate cortex on food craving: an explorative study. *Appetite* 120, 673–678. doi: 10.1016/j.appet.2017.10.034

Leong, S. L., Vanneste, S., Lim, J., Smith, M., Manning, P., and De Ridder, D. (2018a). A randomised, double-blind, placebo-controlled parallel trial of closed-loop infraslow brain training in food addiction. *Sci. Rep*. 8:11659. doi: 10.1038/s41598-018-30181-7

Li, G., Ji, G., Hu, Y., Liu, L., Jin, Q., Zhang, W., et al. (2019). Reduced plasma ghrelin concentrations are associated with decreased brain reactivity to food cues after laparoscopic sleeve gastrectomy. *Psychoneuroendocrinology* 100, 229–236. doi: 10.1016/j.psyneuen.2018.10.022

Mallorquí-Bagué, N., Lozano-Madrid, M., Testa, G., Vintró-Alcaraz, C., Sánchez, I., Riesco, N., et al. (2020). Clinical and neurophysiological correlates of emotion and food craving regulation in patients with anorexia nervosa*. J. Clin. Med*. 9:960. doi: 10.3390/jcm9040960

Meule, A. (2018). Food cravings in food addiction: exploring a potential cut-off value of the food cravings questionnaire-trait-reduced. *Eat. Weight Disord*. EWD 23, 39–43. doi: 10.1007/s40519-017-0452-3

Meule, A., and Kübler, A. (2012). Food cravings in food addiction: the distinct role of positive reinforcement*. Eat. Behav*. 13, 252–255. doi: 10.1016/j.eatbeh.2012.02.001

Meule, A., Freund, R., Skirde, A. K., Vogele, C., and Kubler, A. (2012b). Heart rate variability biofeedback reduces food cravings in high food cravers. *Appl. Psychophysiol. Biofeedback* 37, 241–251. doi: 10.1007/s10484-012-9197-y

Meule, A., Heckel, D., Jurowich, C. F., Vögele, C., and Kübler, A. (2014a). Correlates of food addiction in obese individuals seeking bariatric surgery. *Clin. Obes*. 4, 228–236. doi: 10.1111/cob.12065

Meule, A., Hermann, T., and Kübler, A. (2015). Food addiction in overweight and obese adolescents seeking weight-loss treatment. *Eur. Eat. Disord. Rev. J. Eat. Disord. Assoc*. 23, 193–198. doi: 10.1002/erv.2355

Meule, A., Lutz, A. P. C., Vögele, C., and Kübler, A. (2014b). Impulsive reactions to food-cues predict subsequent food craving. *Eat. Behav*. 15, 99–105. doi: 10.1016/j.eatbeh.2013.10.023

Meule, A., Müller, A., Gearhardt, A. N., and Blechert, J. (2017). German version of the Yale food addiction scale 2.0: prevalence and correlates of ‘food addiction’ in students and obese individuals. *Appetite* 115, 54–61. doi: 10.1016/j.appet.2016.10.003

Meule, A., Skirde, A. K., Freund, R., Vögele, C., and Kübler, A. (2012a). High-calorie food-cues impair working memory performance in high and low food cravers. *Appetite* 59, 264–269. doi: 10.1016/j.appet.2012.05.010

Niemiec, M. A., Boswell, J. F., and Hormes, J. M. (2016). Development and initial validation of the obsessive compulsive eating scale. *Obesity (Silver Spring, Md.)* 24, 1803–1809. doi: 10.1002/oby.21529

Oliveira, E., Kim, H. S., Lacroix, E., de Fátima Vasques, M., Durante, C. R., Pereira, D., et al. (2020). The clinical utility of food addiction: characteristics and psychosocial impairments in a treatment-seeking sample. *Nutrients* 12:3388. doi: 10.3390/nu12113388

Pepino, M. Y., Stein, R. I., Eagon, J. C., and Klein, S. (2014). Bariatric surgery-induced weight loss causes remission of food addiction in extreme obesity. *Obesity (Silver Spring, Md.)* 22, 1792–1798. doi: 10.1002/oby.20797

Polk, S. E., Schulte, E. M., Furman, C. R., and Gearhardt, A. N. (2017). Wanting and liking: separable components in problematic eating behavior? *Appetite* 115, 45–53. doi: 10.1016/j.appet.2016.11.015

Ravichandran, S., Bhatt, R. R., Pandit, B., Osadchiy, V., Alaverdyan, A., Vora, P., et al. (2021). Alterations in reward network functional connectivity are associated with increased food addiction in obese individuals. *Sci. Rep*. 11:3386. doi: 10.1038/s41598-021-83116-0

Schulte, E. M., and Gearhardt, A. N. (2017). Development of the modified Yale food addiction scale version 2.0. *Eur. Eat. Disord. Rev*. 25, 302–308. doi: 10.1002/erv.2515

Schulte, E. M., and Gearhardt, A. N. (2021). Attributes of the food addiction phenotype within overweight and obesity. *Eat. Weight Disord*. EWD 26, 2043–2049. doi: 10.1007/s40519-020-01055-7

Schulte, E. M., Sonneville, K. R., and Gearhardt, A. N. (2019). Subjective experiences of highly processed food consumption in individuals with food addiction. *Psychol. Addict. Behav*. 33, 144–153. doi: 10.1037/adb0000441

Sommer, L. M., Halbeisen, G., Erim, Y., and Paslakis, G. (2021). Two of a kind? Mapping the psychopathological space between obesity with and without binge eating disorder. *Nutrients* 13:3813. doi: 10.3390/nu13113813

Wang, C. H., and Lopez-Fernandez, O. (2019). Shades of foods: prevalence and correlates of food addiction. Aloma Rev. Psicol*. Cienc. l'Educ. l'Esport* 37, 21–34. doi: 10.51698/aloma.2019.37.1.21-34

White, M. A., Whisenhunt, B. L., Williamson, D. A., Greenway, F. L., Netemeyer, R. G., Marney, A., et al. (2002). Development and validation of the food- craving inventory. *Obes. Res*. 10, 107–114. doi: 10.1038/oby.2002.17

Wiedemann, A. A., Carr, M. M., Ivezaj, V., and Barnes, R. D. (2021). Examining the construct validity of food addiction severity specifiers*. Eat. Weight Disord*. EWD 26, 1503–1509. doi: 10.1007/s40519-020-00957-w
